# Burn‐Induced Gut Microbiota Dysbiosis Aggravates Skeletal Muscle Atrophy by Tryptophan‐Kynurenine Mediated AHR Pathway Activation

**DOI:** 10.1002/advs.202409296

**Published:** 2025-02-14

**Authors:** Shan Gao, Yan Leng, Zhen Qiu, Kai Li, Jun Li, Jian Peng, Weiguo Xie, Shaoqing Lei, Zhongyuan Xia

**Affiliations:** ^1^ Department of Anaesthesiology Renmin Hospital of Wuhan University Wuhan Hubei 430060 China; ^2^ Department of Anaesthesiology Tongren Hospital of Wuhan University Wuhan Wuhan Hubei 430060 China; ^3^ Department of Pain Tongren Hospital of Wuhan University Wuhan Hubei 430060 China; ^4^ Department of Burns Tongren Hospital of Wuhan University Wuhan Hubei 430060 China

**Keywords:** burn injury, gut microbiota, gut‐muscle axis, skeletal muscle atrophy, tryptophan

## Abstract

The hypermetabolic response associated with burns is characterized by skeletal muscle atrophy and an increased incidence of disability and death. Significant remodeling of the gut microbiota occurs after severe burn trauma. However, the specific mechanisms by which gut microbiota contribute to burn‐induced muscle atrophy remain unexplored. The results showed that the disruption of the gut microbiota exacerbated skeletal muscle atrophy. Fecal metabolite analysis revealed perturbations, primarily within the tryptophan (Trp) metabolic pathway. Animal models further demonstrated that gut microbiota disorder enhanced the expression of indoleamine 2,3‐dioxygenase 1 (IDO‐1) in the colon, ultimately resulting in Trp depletion and increased kynurenine (Kyn) levels in the serum and skeletal muscle. Excessive colonic Kyn is released into circulation, transported into skeletal muscle cells, and binds to the aryl hydrocarbon receptor (AHR), consequently triggering AHR nuclear translocation and initiating the transcription of skeletal muscle atrophy‐related genes. Notably, serum samples from patients with burns exhibited Trp depletion, and Trp supplementation alleviated skeletal muscle atrophy in rats with burns. This study, for the first time, demonstrates that gut microbiota dysbiosis upregulates colonic IDO‐1, promotes Trp‐Kyn metabolism, and exacerbates burn‐induced skeletal muscle atrophy, suggesting that Trp supplementation may be a potential therapeutic strategy.

## Graphical abstract

This study elucidates a pivotal crosstalk between the gut microbiota and skeletal muscles atrophy induced by burn injuries, a process fueled by the overactivation of IDO‐1 in the colon. This overactivation initiates the metabolism of tryptophan into kynurenine. Subsequently, Kyn is transported into myotubes through SLC38A2 and binds to AHR, and ultimately exacerbates the skeletal muscle atrophy.

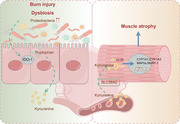

## 1 Introduction

Burns are a major global health problem, causing millions of nonfatal injuries and more than 300 000 deaths annually, as reported by the WHO.^[^ ^1^ ^]^ The uniqueness of severe nonfatal burns lies in the initiation of a hypermetabolic response, prominently manifested as skeletal muscle atrophy and an imbalance in muscle protein synthesis and breakdown.^[^ ^2^ ^]^ This hypermetabolic response induces negative effects on skeletal muscles that persist for months to years and are exacerbated over time by short‐ and long‐term disuse, ultimately leading to burn‐related disabilities and deformities.^[^ ^3^ ^]^ Thus, wound closure alone is insufficient to ensure recovery for burn patients; indeed, the status of the skeletal muscle is paramount to optimizing a patient's quality of life. However, research on the underlying mechanisms that drive skeletal muscle atrophy remains limited, and effective prevention and treatment measures are scarce.

Growing evidence indicates that the gut microbiota interacts with the host to modulate the physiological, behavioral, and genetic processes important for the development and general health of the host.^[^ ^4^, ^5^, ^6^, ^7^ ^]^ As high‐throughput sequencing technology becomes more powerful and precise, our understanding of the significance of changes in the gut microbiota following burns is expanding.^[^ ^8^ ^]^ Burn trauma disrupts the gut microbiota, predominantly through an increase in the abundance of *Proteobacteria* in patients with burns.^[^ ^9^ ^]^ We have previously confirmed this finding in burned rats, where *Proteobacteria* abundance was restored using dietary fiber, which mitigated burn‐induced skeletal muscle atrophy,^[^ ^10^ ^]^ thus, highlighting the critical role of the gut microbiota in this pathological process. However, the mechanisms by which gut microbiota dysbiosis leads to burn‐induced skeletal muscle atrophy remain unclear. Considering that altered gut microbiota contributes to the pathogenesis of muscle atrophy‐related diseases through bacterium‐related metabolites,^[^ ^10^, ^11^, ^12^ ^]^ we conducted an in‐depth investigation to examine the effects of burns on the gut microbiota and derived metabolites, as well as their roles in skeletal muscle status. Our data suggest that burn‐induced dysregulation of the gut microbiota, particularly the abnormal increase in *Proteobacteria*, promotes Trp metabolism in the colon, leading to the accumulation of Kyn and subsequent activation of the AHR pathway in skeletal muscle, which ultimately exacerbates skeletal muscle atrophy.

## 2 Results

### 2.1 Burns Result in Skeletal Muscle Atrophy Accompanied by Gut Microbiota Dysbiosis

**Figure 1:**
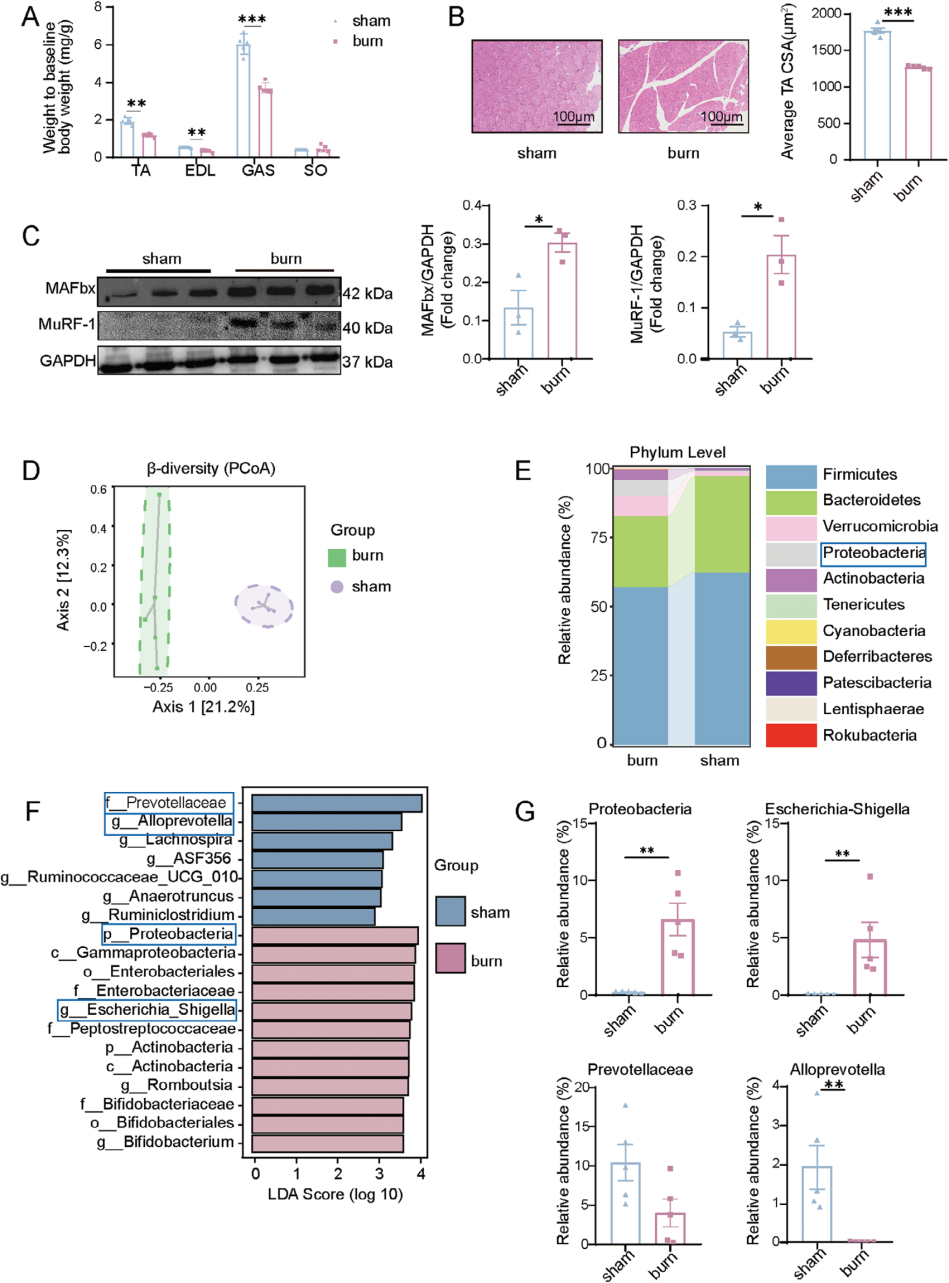
Burn trauma induces skeletal muscle atrophy accompanied by gut microbiota dysbiosis. A) Relative weights of the TA, EDL, GAS, and SO muscles in the burn and sham groups (*n* = 5 per group). B) Representative hematoxylin and eosin (H&E) staining of TA myofiber sections and variation in myofiber CSA (scale bar, 100 µm. *n* = 5 mice per group). C) Relative protein expression of MAFbx and MuRF‐1 in the muscle of burn and sham groups (*n* = 3 per group). D) Principal coordinates analysis (PCoA) score plot assessing fecal microbiota composition in burn and sham rats (*n* = 5 per group). Statistical significance was determined by permutational multivariate analysis of variance using Adonis. E) Relative abundance in the feces at phylum level from burn and sham groups. F) LEfSe analysis showed the specific bacteria differentially expressed in burn and sham groups. G) Fecal contents of burn and sham groups were analyzed for *Proteobacteria*, *Escherichia‐Shigella*, *Prevotellaceae*, and *Alloprevotella* colonization using RT‐qPCR (*n* = 5 per group). All quantitative data were analyzed using an unpaired two‐tailed Student's *t*‐test and are shown as mean ± SEM. ^*^ *p* ≤ 0.05; ^**^ *p* ≤ 0.01; and ^***^ *p* ≤ 0.001.

In a model of third‐degree burns covering 30% of the total body surface area (TBSA), significant reductions were found in the relative weights of the tibialis anterior (TA), extensor digitorum longus (EDL), and gastrocnemius (GAS) muscles (**Figure** **1**A), whereas the relative weights of the liver, spleen, and soleus (SO) remained significantly unchanged (Figure S1A, Supporting Information). Burned rats also showed a significantly decreased TA myofiber cross‐sectional area (CSA) (Figure 1B) and increased expression of muscle atrophy F‐box (MAFbx) and muscle‐specific ring finger protein 1 (MuRF‐1) (Figure 1C; Figure S1B, Supporting Information), suggesting skeletal muscle atrophy.

We then analyzed the effects of burns on the gut microbiota by sequencing 16S rRNA genes. α‐Diversity analysis revealed that burns did not alter gut microbiota diversity (Figure S1C, Supporting Information). However, β‐diversity analysis (using principal coordinates analysis [PCoA]) showed significantly altered composition of the fecal microbiome in the burn group (Figure 1D). We further analyzed the relative abundance of gut microbes at the phylum and genus levels. Specifically, the phyla *Firmicutes* and *Bacteroidetes* were dominant in both the sham and burn groups, whereas *Verrucomicrobia* and *Proteobacteria* were enriched in the burn group (Figure 1E), and the genera *Lactobacillus*, *Bacteroides*, and *Escherichia‐Shigella* were particularly enriched (Figure S1D, Supporting Information). Further analysis revealed the top 20 most enriched bacterial species (Figure S1E, Supporting Information). The linear discriminant analysis (LDA) effect size (LEfSe) analysis revealed that the taxonomic chain *Proteobacteria–Gammaproteobacteria–Enterobacteriales–Enterobacteriaceae–Escherichia‐Shigella* contributed the most to the difference in gut microbiota in burned rats (Figure 1F). Notably, burned rats experienced a marked increase in the relative abundance of the phylum *Proteobacteria* and the genus *Escherichia‐Shigella* (a species within *Proteobacteria* operational taxonomic unit sequences), with a slight decrease in that of the genus *Alloprevotella* (Figure 1G). Further analysis revealed that burn‐induced alterations in the gut microbiota were negatively correlated with muscle weight (TA/EDL/GAS), specifically indicating that the relative abundance of *Proteobacteria* was inversely correlated with muscle weight (Figure S1F,G, Supporting Information). These findings indicate that burns altered the composition of the gut microbiota, particularly by expanding the *Proteobacteria* phylum.

### 2.2 Altered Gut Microbiota Contributes to Burn‐Induced Skeletal Muscle Atrophy

**Figure 2:**
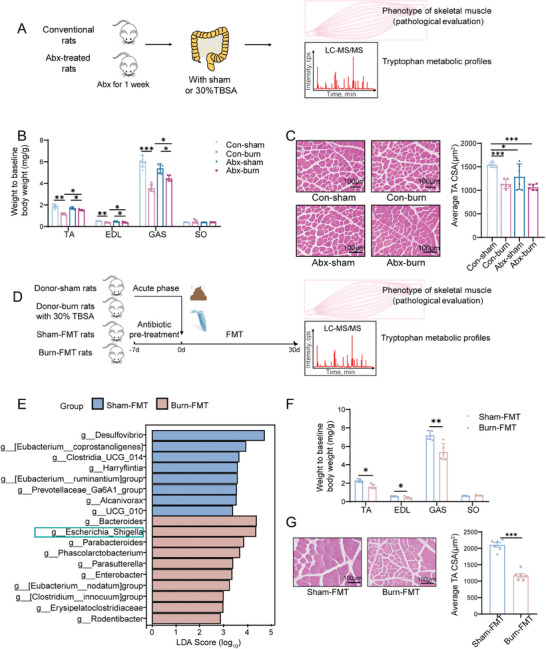
Altered gut microbiota highly correlates with skeletal muscle atrophy. A) Conventional (Con) rats and pseudo‐sterile rats, with gut microbiota depleted by antibiotics (Abx), underwent a sham or 30% TBSA burn, and tryptophan metabolic profiles and/or the degree of skeletal muscle atrophy were measured. B) Relative weights of TA, EDL, GAS, and SO muscles in Con‐burn, Con‐sham, Abx‐burn, and Abx‐sham groups (*n* = 5 per group). C) Representative H&E staining of TA myofiber sections and variation in myofiber CSA in Con‐burn, Con‐sham, Abx‐burn, and Abx‐sham groups (scale bar, 100 µm. *n* = 6 per group). D) Abx‐rats were transplanted intragastrically for 30 days with the feces collected from donor rats that underwent a sham (Donor‐sham group) or 30% TBSA burn (Donor‐burn group), and the degree of skeletal muscle atrophy and Trp metabolic profiles were evaluated. E) LEfSe analysis showed the specific bacteria differentially expressed in burn‐FMT and sham‐FMT groups. F) Relative weights of TA, EDL, GAS, and SO muscles in burn‐FMT and sham‐FMT groups (*n* = 5 per group). G) Representative H&E staining of TA myofiber sections and variation in myofiber CSA in burn‐FMT and sham‐FMT groups (scale bar, 100 µm. *n* = 6 per group). All quantitative data, except (B), were analyzed using an unpaired two‐tailed Student's *t*‐test and are shown as mean ± SEM. The data of (B) were analyzed using one‐way ANOVA. ^*^ *p* ≤ 0.05; ^**^ *p* ≤ 0.01; and ^***^ *p* ≤ 0.001.

To explore the role of alterations in the gut microbiota in burn‐induced skeletal muscle atrophy, we first established an antibiotic pseudo‐germ‐free model according to the protocol shown in **Figure** **2**A. Rats were treated with an antibiotic cocktail (Abx) for 1 week to deplete the commensal gut microbiota (Figure S2A, Supporting Information). These microbiota‐depleted rats had less burn‐induced skeletal muscle atrophy, as indicated by reduced loss of muscle (TA, EDL, and GAS) weight and myofiber CSA (Figure 2B,C), as well as decreased expression of the atrophy markers MAFbx and MuRF‐1 (Figure S2B,C, Supporting Information).

We then established a fecal microbiota transplantation (FMT) model (Figure 2D) to confirm gut microbiota abnormality‐induced skeletal muscle atrophy. After 30 days of FMT, the recipient rats that received feces from the burned rats displayed an increased abundance of the phyla *Firmicutes, Bacteroidetes*, *Verrucomicrobia*, and *Proteobacteria* (Figure S2D, Supporting Information) and the genus *Escherichia‐Shigella* in the colon (Figure S2E, Supporting Information). Notably, LEfSe analysis showed *Escherichia‐Shigella* remained the dominant genus enriched in these recipient rats (burn‐FMT group) (Figure 2E), which had decreased skeletal muscle weight and myofiber CSA, as well as increased expression of MAFbx and MuRF‐1 (Figure 2F,G; Figure S2F,G, Supporting Information). These findings confirm that altered gut microbiota, particularly the increased abundance of *Proteobacteria* and *Escherichia‐ Shigella*, contributed to burn‐induced muscle atrophy.

### 2.3 Burns Alter Gut Microbiota‐Related Trp Metabolic Profile

**Figure 3:**
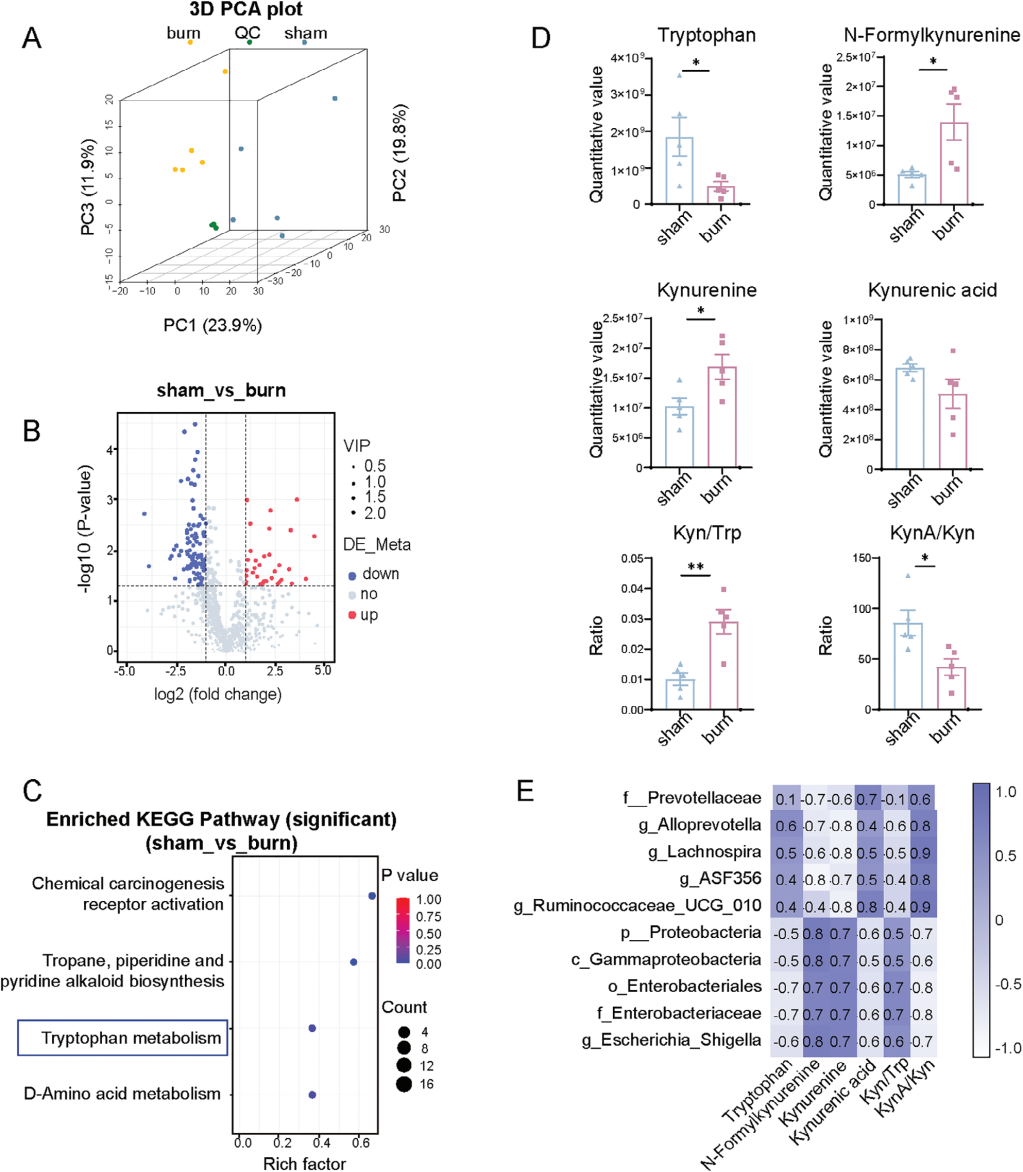
Burns alter gut microbiota‐related Trp metabolic profile. A) The 3D PCA image analysis was conducted on fecal samples, comparing sham and burn groups (*n* = 5 per group). B) A volcano plot was generated to compare different metabolites in the burn group versus the sham group. Up‐regulated metabolites (red) and the down‐regulated metabolites (green) with variable importance in projection (VIP) >1 with a fold change <0.5 or >2 and *p *< 0.05. C) KEGG analysis of metabolic pathways enriched for differential metabolites obtained from the volcano plot analysis. D) Quantitative values of Trp, N‐Kyn, Kyn, KynA, Kyn to Trp (Kyn/Trp) ratio, and KynA to Kyn (KynA/Kyn) ratio were evaluated in fecal samples of the sham and burn groups (*n* = 5 per group). E) Pearson correlation heatmap analysis was performed at the representative LDA >3 microbial genera and Trp‐derived metabolites in fecal samples, comparing the sham and burn groups. The number within and the color of each cell indicates the pearson correlation coefficient and directionality (blue, positive correlation; white, negative correlation). All quantitative data were analyzed using an unpaired two‐tailed Student's *t*‐test and are shown as mean ± SEM. ^*^ *p* ≤ 0.05 and ^**^ *p* ≤ 0.01.

We explored the fecal microbial metabolites using liquid chromatography‐mass spectrometry (LC‐MS). Three‐dimensional principal component analysis (3D PCA) showed good quality control with clearly distinguishable metabolites between the sham and burn groups (**Figure** **3**A). In the positive‐ion mode, 34 metabolites were upregulated and 102 were downregulated in the feces of burned rats (Figure 3B). The Kyoto Encyclopedia of Genes and Genomes (KEGG) analysis of these metabolites showed four enriched pathways, of which the Trp metabolism pathway is mainly involved in the regulation of skeletal muscle weight (Figure 3C).^[^ ^13^, ^14^, ^15^ ^]^ Trp metabolism proceeds via three pathways: the Kyn pathway facilitated by IDO‐1, the serotonin pathway, and the indole pathway.^[^ ^16^ ^]^ Clustered image mapping further revealed distinct metabolite patterns, which were more inclined to convert to the Trp‐Kyn pathway in burned rats (Figure S3A–C, Supporting Information). Furthermore, we detected Trp metabolic profiles in the serum and skeletal muscle and found no differences in metabolites related to the indole or serotonin pathways (Figure S3D,E, Supporting Information). Quantitative analysis of Trp‐derived metabolites revealed decreased Trp levels, increased N‐formylkynurenine (N‐Kyn) and Kyn levels, and an elevated ratio of Kyn to Trp (a proxy for the activity of the major Trp‐degrading enzyme IDO‐1)^[^ ^17^ ^]^ in the feces of burned rats (Figure 3D). However, the ratio of kynurenic acid (KynA) to Kyn (a proxy for kynurenine aminotransferase activity) was decreased. Collectively, these results suggest that burns reprogram Trp metabolism via the Kyn pathway.

We performed pearson's correlation analysis of Trp‐derived metabolites and altered gut microbiota identified by LEfSe analysis. The burn‐induced alterations in the gut microbiota of *Proteobacteria–Gammaproteobacteria–Enterobacteriales–Enterobacteriaceae–Escherichia‐Shigella* had a positive correlation with the concentrations of N‐Kyn and Kyn, as well as the ratio of Kyn/Trp (Figure 3E), suggesting that disrupted phylum *Proteobacteria* is closely related to altered Trp metabolism in burns.

### 2.4 Dysregulation of Trp‐Kyn Metabolism in Skeletal Muscle Induced by Gut Dysbiosis is Attributable to Colonic IDO‐1 Overactivation

**Figure 4:**
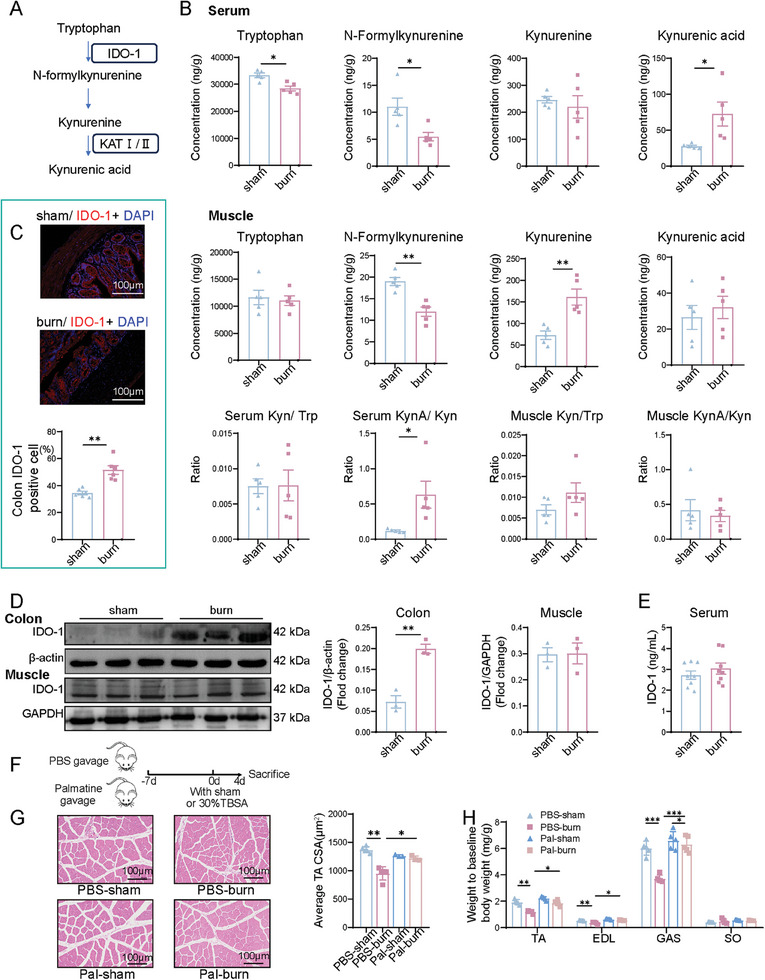
Hyperactivated IDO‐1 in colonocytes mediates the gut dysbiosis‐induced dysregulation of serum and skeletal muscle Trp‐Kyn metabolism and is associated with muscle atrophy. A) Outline of the Trp‐derived Kyn metabolic pathway. B) Levels of Trp, N‐Kyn, Kyn, KynA, Kyn/Trp ratio, and KynA/Kyn ratio were measured in serum and muscle tissue, comparing the sham and burn groups (*n* = 5 per group). C) IF staining of IDO‐1 in the colon from sham and burn groups at 4 days post‐burn, with IDO‐1‐positive cell analysis (scale bar, 100 µm. *n* = 6 per group). D) Relative IDO‐1 protein expression in colon and muscle tissue from sham and burn groups (*n* = 3 per group). E) Enzyme‐linked immunosorbent assay (ELISA) of serum IDO‐1 levels from sham and burn groups (*n* = 8 per group). F) Rats received palmatine (100 mg kg^−1^ d^−1^) or phosphate‐buffered saline (PBS) via gastric gavage for 7 days prior to sham or 30% TBSA burn. G) Representative H&E staining of TA myofiber sections and CSA variation in PBS‐sham, PBS‐burn, Pal‐burn, and Pal‐sham groups at 4 days post‐burn. Five representative views were randomly selected to calculate statistical significance (scale bar, 100 µm. *n* = 5 per group). H) Relative weights of the TA, EDL, GAS, and SO muscles in PBS‐burn, PBS‐sham, Pal‐burn, and Pal‐sham groups (*n* = 5 per group). All quantitative data, except (H), were analyzed using an unpaired two‐tailed Student's *t*‐test and are shown as mean ± SEM. The data of (H) were analyzed using one‐way ANOVA. ^*^ *p* ≤ 0.05; ^**^ *p* ≤ 0.01; and ^***^ *p* ≤ 0.001.

As shown in **Figure** **4**A, Trp metabolism via the Kyn pathway is mediated by the rate‐limiting enzyme, IDO‐1, resulting in the generation of Kyn and its downstream products. To assess the potential impact of altered gut microbiota on the levels of Trp‐derived metabolites, high‐performance liquid chromatography‐tandem mass spectrometry (HPLC‐MS/MS) analysis was conducted on the serum and skeletal muscle. In the serum of burned rats, Trp and N‐Kyn levels were significantly decreased, accompanied by increased KynA levels. The skeletal muscles of burned rats also exhibited decreased N‐Kyn levels and increased Kyn levels (Figure 4B) but Trp levels remained unchanged, possibly due to the breakdown of skeletal muscle proteins into amino acids to fulfill the requirements for burn wound healing.^[^ ^18^ ^]^ Consequently, potential alterations in Trp levels in the skeletal muscles were obscured, concealing a reduction in Trp uptake by the skeletal muscles. Furthermore, correlation analysis showed that muscle Kyn levels were negatively correlated with muscle weight (for TA, EDL, and GAS) (Figure S4A, Supporting Information).

IDO‐1 is expressed not only in the colon but also in smaller quantities in serum and skeletal muscle.^[^ ^19^ ^]^ Kyn, which accumulates in the muscle tissue, is likely transported into skeletal muscle cells via the large neutral amino acid transporter solute carrier family 38 member 2 (SLC38A2).^[^ ^20^ ^]^ Furthermore, skeletal muscle cells contribute to Kyn production through Trp metabolism.^[^ ^21^ ^]^ To elucidate the origin of Kyn in the muscle, we examined the expression of IDO‐1 in the colon, serum, and skeletal muscle. Immunofluorescence (IF) staining revealed an increased number of IDO‐1‐positive cells in the colons of burned rats (Figure 4C). Further analysis confirmed upregulated IDO‐1 expression in the colon but without significant alterations in the level in the serum and skeletal muscle of burned rats (Figure 4D,E; Figure S4B, Supporting Information). These results collectively suggest that the increased Kyn in the skeletal muscle originates primarily from colonic IDO‐1‐catalyzed Trp production rather than from Trp metabolism within the skeletal muscle.

To further confirm the influence of colonic IDO‐1 on skeletal muscle, palmatine (Pal), a potent IDO‐1 inhibitor, was used to inhibit IDO‐1 activity in burned rats (see the protocol in Figure 4F). Pal effectively inhibited IDO‐1 expression in the colon (Figure S4C,D, Supporting Information) and reversed the burn‐induced decrease in myofiber CSA (Figure 4G) and muscle (TA, EDL, and GAS) weights (Figure 4H), indicating that the inhibition of colonic IDO‐1 can attenuate burn‐induced skeletal muscle atrophy.

Collectively, these findings suggest that the gut microbiota promotes programmed metabolism of Trp to Kyn, exacerbating skeletal muscle atrophy post‐burn.

### 2.5 Altered Gut Microbiota is Essential for Burn‐Induced Abnormal Trp‐Derived Metabolites via Colonic IDO‐1 in Skeletal Muscle

**Figure 5:**
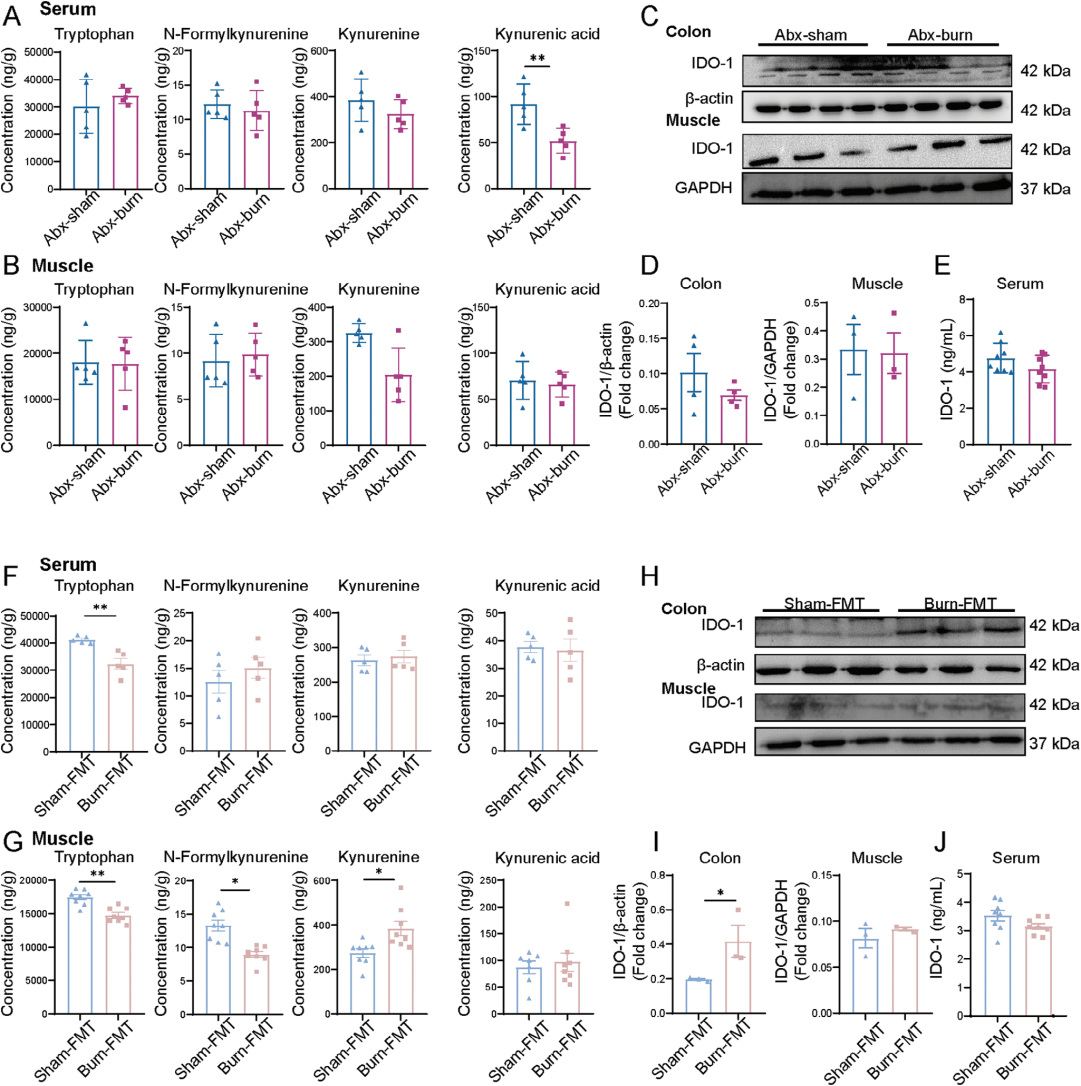
The causal role of gut microbiota in mediating burn‐induced Trp depletion and Kyn accumulation in serum and skeletal muscle. A,B) Serum and muscle tissue levels of Trp, N‐ Kyn, Kyn, and KynA in Abx‐sham and Abx‐burn groups (*n* = 5 per group). C,D) Relative IDO‐1 protein expression in colon and muscle from Abx‐sham and Abx‐burn groups (*n* = 4 per group). E) ELISA of serum IDO‐1 levels from Abx‐sham and Abx‐burn groups at 4 days after burn (*n* = 8 per group). F,G) Serum and muscle tissue levels of Trp, N‐Kyn, Kyn, and KynA in sham‐FMT and burn‐FMT groups (*n* = 5 per group in F, *n* = 8 per group in G). H,I) Relative IDO‐1 protein expression in colon and muscle tissue from sham‐FMT and burn‐FMT groups (*n* = 3 per group). J) ELISA of serum IDO‐1 levels from sham‐FMT and burn‐FMT groups (*n* = 8 per group). All quantitative data were analyzed using an unpaired two‐tailed Student's *t*‐test and are shown as mean ± SEM. ^*^ *p* ≤ 0.05 and ^**^ *p* ≤ 0.01.

We further confirmed the role of altered gut microbiota in colonic IDO‐1 mediated Trp‐Kyn metabolic disorders of the skeletal muscle in an antibiotic pseudo‐septic model and an FMT model. After treatment with Abx to deplete the commensal gut microbiota, there were no significant changes in Trp‐Kyn metabolites in either the serum or skeletal muscle between sham and burned rats, except for a reduction in KynA levels (**Figure** **5**A,B; Figure S5A, Supporting Information). Furthermore, there was no significant change in IDO‐1 expression in the colon, skeletal muscle, or serum (Figure 5C–E; Figure S5B,C, Supporting Information). However, after 30 days of FMT, recipient rats that received feces from burned rats exhibited marked reductions in serum and skeletal muscle Trp levels, with increased skeletal muscle Kyn levels (Figure 5F,G; Figure S5D, Supporting Information), consistent with the rise in Kyn in the skeletal muscle of burned rats (Figure 4B). Additionally, recipient rats that received feces from burned rats displayed higher colonic IDO‐1 expression than those that received feces from sham rats. No significant difference was observed in IDO‐1 expression in the skeletal muscle and serum between the groups (Figure 5H–J; Figure S5E,F, Supporting Information). Collectively, these findings highlight the pivotal role of altered gut microbiota in mediating abnormal Trp‐Kyn metabolism in post‐burn skeletal muscles, primarily through the upregulation of colonic IDO‐1.

### 2.6 Kyn Induces Proteolysis in L6 Myotubes Via the Activation of the AHR Pathway

**Figure 6:**
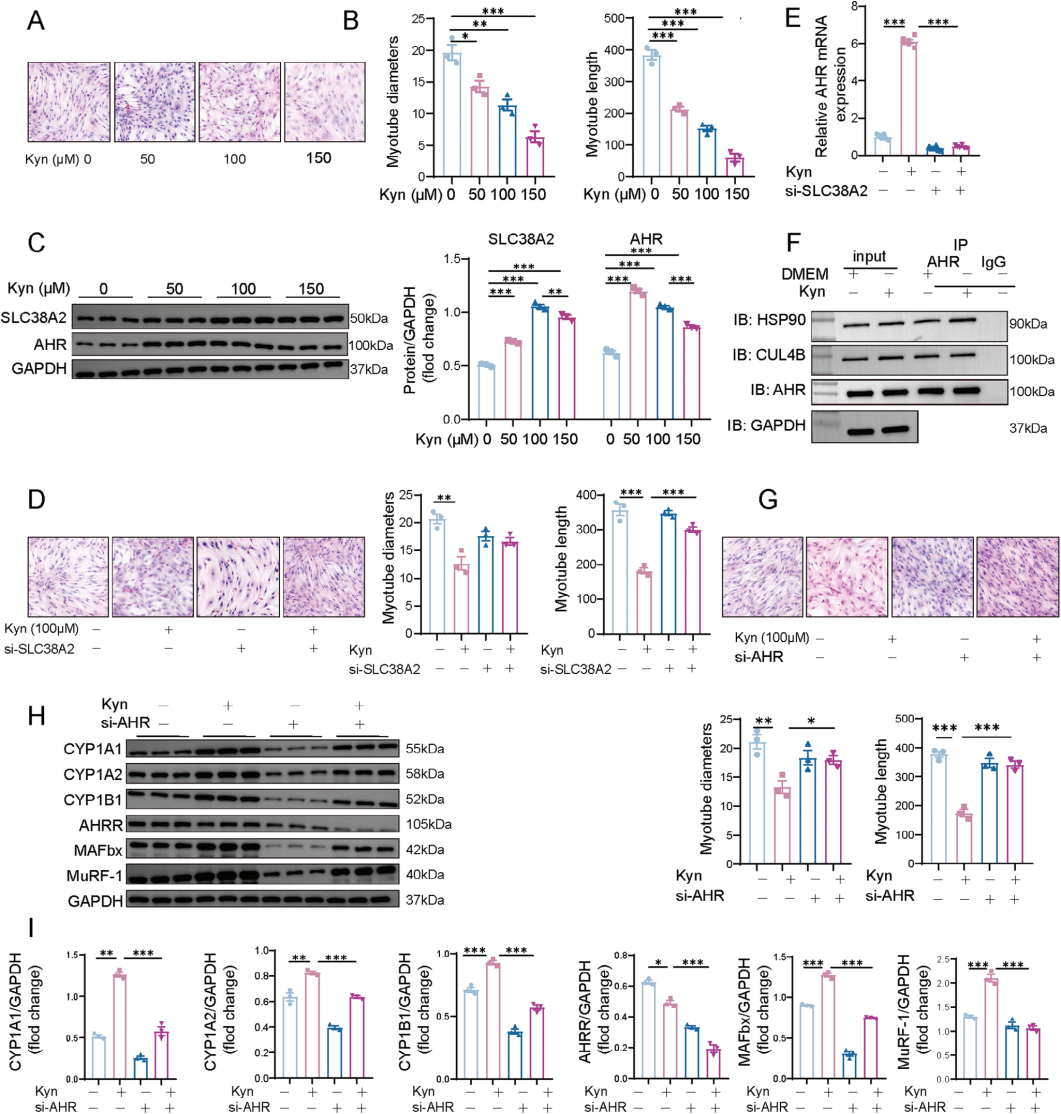
Kyn induces L6 myotube proteolysis through the activation of the AHR pathway. A) L6 myotubes were treated with Kyn (50, 100, or 150 µmol) or vehicle for 24 h. Myotube morphology was examined via H&E staining (scale bar, 50 µm). B) Effects of Kyn on the diameter and length of L6 myotubes (*n* = 3 per group). C) Changes in SLC38A2 and AHR protein expression in L6 myotubes treated with Kyn (50, 100, or 150 µmol) or vehicle for 24 h (*n* = 3 per group). D) L6 myotubes pre‐treated with SLC38A2 siRNA (100 nmol) and incubated with Kyn (100 µmol). H&E staining was used to examine the myotube morphology and effects of different treatments on the diameter and length of L6 myotubes (scale bar, 50 µm. *n* = 3 per group). E) Bar graph showing RT‐qPCR quantification of *AHR* gene expression levels in L6 myotubes transfected with SCL38A2 siRNAs or vehicle (*n* = 3 per group). F) L6 myotubes were pretreated with DMSO or Kyn supplementation. The expression of AHR, HSP90, Cul4B, and GAPDH was determined by western blot analysis (input). Immunoprecipitation was performed with AHR or IgG antibody. HSP90, Cul4B, HSP90‐bound AHR, and Cul4B‐bound AHR were determined by western blot analysis (IP; *n* = 3). G) L6 myotubes pre‐treated with AHR siRNA (100 nmol) and incubated with Kyn (100 µmol). H&E staining was used to examine the myotube morphology and effects of different treatments on the diameter and length of L6 myotubes (scale bar, 50 µm. *n* = 3 per group). H,I) Changes in CYP1A1, CYP1A2, CYP1B1, AHRR, MAFbx, and MuRF‐1 protein expression in L6 myotubes transfected with AHR siRNAs or vehicle (*n* = 3 per group). All quantitative data were analyzed using one‐way ANOVA and are shown as mean ± SEM. ^*^ *p* ≤ 0.05; ^**^ *p* ≤ 0.01; and ^***^ *p* ≤ 0.001.

L6 myoblasts were differentiated into myotubes (Figure S6A,B, Supporting Information), and hematoxylin and eosin staining revealed a dose‐dependent reduction in both myotube diameter and length after Kyn exposure (**Figure** **6**A,B). Considering that SLC38A2 and AHR can be expressed in L6 myotubes, with SLC38A2 being the sole amino acid transporter consistently found in skeletal muscle,^[^ ^20^ ^]^ we detected a significant increase in the mRNA and protein levels of SCL38A2 and AHR after Kyn treatment for 24 h (Figure 6C; Figure S6C, Supporting Information). This was accompanied by decreased myotube length and upregulated AHR expression, which was significantly attenuated by SLC38A2 knockdown (Figure 6D,E; Figure S6D, Supporting Information). To investigate the cellular signaling cascade triggered by the intracellular binding of Kyn to AHR, a co‐immunoprecipitation (COIP) assay was conducted in myotubes, which showed that AHR was co‐immunoprecipitated with heat shock protein 90 (HSP90) but not with cullin‐4B (Cul4B) (Figure 6F). We further verified the role of AHR in myotube atrophy. AHR knockdown mitigated Kyn‐induced atrophy, as evidenced by increased myotube diameter and length (Figure 6G; Figure S6E, Supporting Information). Furthermore, AHR knockdown attenuated Kyn‐induced upregulation of CYP1A1, CYP1A2, CYP1B1, MAFbx, and MuRF‐1 expression, as well as Kyn‐induced downregulation of AHRR expression (Figure 6H,I; Figure S6F, Supporting Information). These findings indicate that Kyn, facilitated by SLC38A2‐mediated cellular entry, binds to AHR to regulate myotube atrophy.

### 2.7 Trp Metabolism is Disordered in Patients with Burns

**Figure 7:**
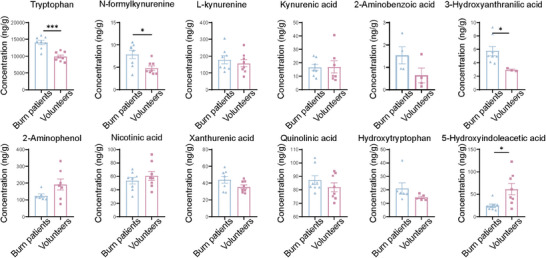
Analyses of serum from patients with burns reveal disturbances in Trp metabolism, as exhibited by Trp depletion (*n* = 8 per group). All quantitative data were analyzed using an unpaired two‐tailed Student's *t*‐test and are shown as mean ± SEM. ^*^ *p* ≤ 0.05 and ^***^ *p* ≤ 0.001.

We also investigated Trp metabolites in patients with burns. Table S1 (Supporting Information) presents the general conditions of the patients with burns and healthy volunteers. A significant reduction in the third lumbar skeletal muscle index was observed in patients with burns. HPLC‐MS/MS analysis showed that serum Trp decreased significantly in patients with burns, accompanied by reduced levels of N‐Kyn and 3‐hydroxyanthranilic acid and increased levels of 5‐hydroxyindoleacetic acid (**Figure** **7**), indicating that burns caused disorders in Trp metabolism.

### 2.8 Trp‐Enriched Diet Ameliorates Skeletal Muscle Atrophy in Burned Rats

**Figure 8:**
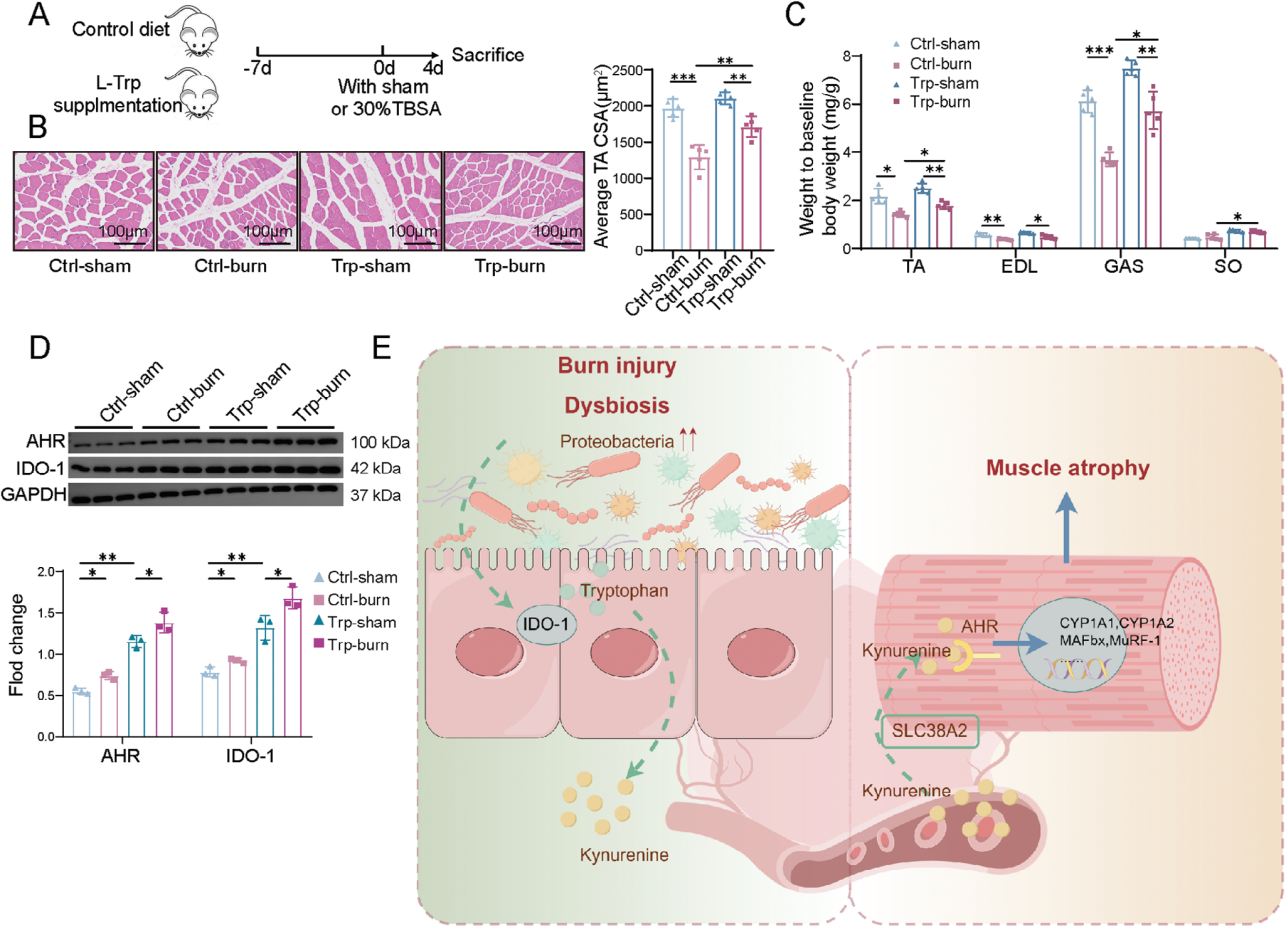
Trp‐supplemented diet improves skeletal muscle atrophy in burned rats. A) Schematic of the Trp‐supplementation experiment in rats with or without burn trauma. B) Representative H&E staining of TA myofiber sections and variation in CSA in different groups at 4 d post‐burn. Five representative views were randomly selected to calculate statistical significance (scale bar, 100 µm. *n* = 3 per group). C) Relative weights of the TA, EDL, GAS, and SO muscles (*n* = 3 per group). D) Western blot analysis showed the expression of AHR and IDO‐1in colon (*n* = 3 per group). E) Schematic representation of microbial dysbiosis, featuring *Proteobacteria* proliferation, that triggers IDO‐1 expression in the colon. This activation leads to the metabolism of Trp to Kyn, which increases in the blood and accumulates in the skeletal muscle. Kyn is translocated into myotubes via SLC38A2 and binds to AHR, activating the AHR signaling pathway, thereby exacerbating burn‐induced skeletal muscle atrophy. All quantitative data were analyzed using one‐way ANOVA. ^*^ *p* ≤ 0.05; ^**^ *p* ≤ 0.01; and ^***^ *p* ≤ 0.001.

To determine whether Trp supplementation could improve burn‐induced muscle wasting, rats were fed a Trp‐enriched diet for 7 days (**Figure** **8**A). After the following 7 days of PBS or Trp supplementation, the burn induced skeletal muscle atrophy were reversed by Trp, manifesting as the increased myofiber CSA and muscle weight in the Trp‐burn group compared with the Ctrl‐burn group (Figure 8B,C; Figure S6G, Supporting Information). Additionally, Trp supplementation elevated the expression levels of AHR and IDO‐1 in the colon (Figure 8D). These findings suggest that Trp supplementation can prevent muscle mass loss caused by gut‐muscle axis dysfunction after burns.

## 3 Discussion

This is the first study to demonstrate that burn‐induced dysbiosis of the gut microbiota exacerbates the progression of skeletal muscle atrophy via Trp metabolism disorders. Specifically, post‐burn microbiota dysbiosis, particularly the increased abundance of *Proteobacteria*, induces colonic IDO‐1 hyperactivation, which facilitates the metabolic depletion of Trp and the accumulation of Kyn in the colon. Excessive colonic Kyn is released into circulation, transported into skeletal muscle cells via SLC38A2, and binds to AHR, activating the canonical AHR pathway. This activation triggers the translocation of AHR into the nucleus, where it initiates the transcription of downstream target genes, ultimately augmenting the transcription of skeletal muscle atrophy‐related proteins (Figure 8D). Importantly, patients with burns exhibit reduced circulating Trp levels. Oral Trp supplementation alleviated skeletal muscle atrophy in rats with burns. Taken together, these results reveal the central role and underlying mechanisms of the gut microbiota as regulators of the potential crosstalk between the gut and muscles induced by burn injuries.

Burns promptly disrupt gut microbiota, particularly increasing the proliferation of opportunistic pathogens.^[^ ^22^ ^]^ Our study focused on the specific impact of burns on the gut microbiota and found a significant increase in the abundance of *Proteobacteria* and *Escherichia‐Shigella* in the colons of burned rats. Under normal physiological conditions, the abundance of *Proteobacteria* is typically maintained below 10% in the colon,^[^ ^23^ ^]^ and its marked elevation indicates gut dysbiosis.^[^ ^24^ ^]^ To investigate the pivotal role of gut microbiota in burn‐induced skeletal muscle catabolism, we conducted research using antibiotic pseudo‐septic and FMT models, providing clear evidence supporting a causal relationship between gut microbiota dysbiosis and muscle atrophy. Although germ‐free mice exhibited skeletal muscle atrophy, decreased protein expression levels at the neuromuscular junction, and diminished transcription of genes related to skeletal muscle growth and mitochondrial function,^[^ ^25^ ^]^ our findings revealed that burns did not further exacerbate skeletal muscle atrophy after antibiotic‐mediated depletion of the gut microbiota. Further investigation revealed that FMT from burn‐injured rats exacerbated muscle atrophy in the recipient animals, indicating that gut microbiota dysregulation adversely affects skeletal muscle health. Specifically, the results showed a significant increase in the abundance of *Proteobacteria* and *Escherichia‐Shigella* in the colons of recipient rats, with *Escherichia‐Shigella* remaining the key differentially expressed bacterial species enriched in the recipient group. This aberrant gut microbiota alters the metabolism of the host skeletal muscle, primarily manifesting as reduced skeletal muscle mass, decreased myofiber CSA, and increased expression of *MAFbx* and *MuRF‐1*, master genes of muscle atrophy.^[^ ^26^ ^]^ Conversely, transplanting feces from healthy aged mice into young germ‐free mice increased the body weight and skeletal muscle mass of the recipient mice.^[^ ^27^ ^]^ These observations highlight the crucial role of abnormalities in the gut microbiota in driving post‐burn skeletal muscle atrophy. Consistent with a recent study,^[^ ^28^ ^]^ we highlighted the vital role of gut microbiota composition and diversity in skeletal muscle metabolism and function, a phenomenon known as the gut‐muscle axis.

Several studies have emphasized the critical function of bioactive metabolites derived from gut microbiota in regulating host physiological processes.^[^ ^29^, ^30^ ^]^ However, the specific metabolic derivatives of the gut microbiota that serve as key molecular mediators of skeletal muscle atrophy remain unclear. Therefore, we analyzed fecal metabolites and found that the differential metabolites were primarily concentrated within four metabolic pathways (Figure 3C), of which the Trp metabolic pathway is mainly involved in the regulation of skeletal muscle weight.^[^ ^13^, ^14^, ^15^ ^]^ Trp is a potential substance that connects the microbiota to the host physiology and is a precursor of various bioactive compounds that affect host metabolism, inflammation, and oxidative stress responses.^[^ ^31^ ^]^ Recent studies have linked key metabolite profiles in skeletal muscles, particularly the Kyn pathway of Trp, NAD^+^, and acylcarnitine, with phenotypic features of healthy aging, such as cardiorespiratory exercise and mitochondrial function.^[^ ^32^ ^]^ Furthermore, significant differences exist in the levels of Trp, Kyn, KynA, quinolinic acid, and NAD^+^ in skeletal muscles between older and younger adults.^[^ ^17^ ^]^ Physical exercise modulates Trp‐Kyn metabolism in skeletal muscles, thereby altering the concentrations of Kyn pathway metabolites.^[^ ^14^ ^]^ These findings underscore the pivotal role of Trp metabolism in skeletal muscles.

Trp metabolism proceeds via three pathways: the Kyn pathway facilitated by IDO‐1, the 5‐hydroxytryptamine production pathway, and the transformation of Trp into AHR ligands through the indole pathway.^[^ ^33^ ^]^ To elucidate the specific pathway through which burns affect Trp metabolism, we analyzed the Trp metabolic profile in the serum and skeletal muscle of burned rats and found no differences in metabolites related to the indole or serotonin pathways, but significantly decreased serum Trp levels and increased Kyn levels. Consistent with this finding, the patients with burns in our study also showed a significant reduction in serum Trp levels. Additionally, patients with sepsis and muscle atrophy had reduced serum levels of Trp and elevated levels of Kyn.^[^ ^34^ ^]^ Collectively, our data demonstrate that burns may reprogram Trp metabolism via the Kyn pathway.

Trp metabolism via the Kyn pathway involves the initial conversion of Trp to Kyn via IDO‐1, which is the rate‐limiting step. Colonic IDO‐1 plays a pivotal role in the interaction between the host and its microbiota and exhibits remarkable regulatory activity. Our findings indicate that alterations in the gut microbiota after burns lead to the upregulation of IDO‐1 expression in the colon. This upregulation enhances Trp metabolism, resulting in a significant increase in Kyn in skeletal muscle. Notably, the elimination of the gut microbiota did not affect colonic IDO‐1 expression, and no significant differences were observed in the serum and skeletal muscle Trp‐Kyn profiles. However, transplantation of gut microbiota from burned rats upregulated IDO‐1 expression in the colon of recipient rats, resulting in Trp depletion and Kyn accumulation in skeletal muscle. Importantly, inhibition of IDO‐1 in the colon by Pal alleviated post‐burn skeletal muscle atrophy. It has been shown that a high‐fat diet can increase the abundance of *Proteobacteria*, thereby inducing the expression level of colonic IDO‐1.^[^ ^24^ ^]^ Interestingly, whey protein isolate gavage reduced the abundance of *Proteobacteria* in mice and downregulated colonic IDO‐1.^[^ ^35^ ^]^ These observations suggest that altered gut microbiota is a key factor in abnormal Trp metabolism, affecting overall Trp metabolism in the body through the modulation of colonic IDO‐1 expression.

Notably, our study showed that in skeletal muscle cells, Kyn binding to AHR activates the canonical AHR pathway, ultimately leading to the transcription of downstream target genes and the breakdown of muscle cell proteins. As the only amino acid transporter consistently found in skeletal muscle,^[^ ^36^ ^]^ SLC38A2 transports Kyn into myotubes, which then bind to the endogenous ligand AHR. This interaction is manifested by a reduction in myotube length and diameter, whereas SLC38A2 knockdown resulted in longer myotubes. A COIP assay showed that Kyn binds to HSP90 in skeletal muscle cells, but not to Cul4B. However, in vascular smooth muscle cells, Kyn promotes the binding and ubiquitination of AHR with Cul4B through a nonclassical pathway, resulting in proteasomal degradation through an AHR‐dependent nongenomic pathway.^[^ ^31^, ^32^ ^]^ Logically, the reduced oxidative capacity of muscles and the degeneration of neuromuscular junctions resulting from chronic AHR activation also contribute to muscle atrophy. Our study presents a novel perspective, demonstrating that Kyn binding to AHR facilitates its translocation into the nucleus, ultimately leading to the expression of genes associated with muscle atrophy. Notably, AHR knockdown in myotubes led to an increase in myotube diameter and length, indicating a reversal of this atrophy‐promoting mechanism. These findings serve as a basis for future research that seeks to unravel the potential involvement of AHR in diseases associated with muscle atrophy.

To explore the role of nutritional supplementation in preventing burn‐induced muscle atrophy, we found that Trp supplementation had a profound positive effect on the CSA of myofibers after burn injury. Trp promotes the expression of IDO‐1 and AHR, likely due to the fact that increased levels of the metabolic substrate Trp compensatively enhance the expression of its key metabolic enzymes, IDO‐1 and AHR.^[^ ^37^ ^]^ Previous studies have established that Trp substantially affects muscle mass, with Trp‐deficient animals exhibiting attenuated levels of growth hormone (GH) and marked muscle atrophy, which are downstream consequences of diminished GH/IGF1 signaling pathways.^[^ ^38^, ^39^ ^]^ Mice fed a Trp‐enriched diet displayed increased lean body mass and their muscles exhibited significant upregulation of molecules within the mTOR/eIF4E/p70S6K signaling pathway, a key regulatory cascade involved in protein synthesis and muscle hypertrophy.^[^ ^40^ ^]^ Although the precise mechanisms underlying these observations remain to be fully elucidated, the current evidence highlights the pressing necessity for detailed molecular and cellular biological follow‐up studies to decipher these complex phenomena.

## 4 Conclusion

This study revealed a new mechanistic link between gut microbiota, Trp metabolites, AHR signaling, and skeletal muscle atrophy. Our current observations highlight the dynamic regulation of IDO‐1 by the gut microbiota. As a key participant in Trp metabolism, IDO‐1 activity is directly related to augmented Kyn levels in the skeletal muscle microenvironment. This augmentation triggers a series of events that specifically upregulate the expression of atrophy‐associated genes. This finding not only enhances our understanding of the pathological pathways of skeletal muscle but also opens a novel avenue for managing skeletal muscle health through modulation of the gut microbiota‐Trp metabolism axis.

## 5 Experimental Section

### Ethics Approval

All animal experiments were conducted in accordance with the ethical guidelines set by the Animal Ethics Committee of Tongren Hospital, Wuhan University, prioritizing animal welfare per National Institutes of Health regulations. Male Sprague‐Dawley rats (6–8 weeks old, weighing 180–210 grams) were obtained from Sanxia University, China (certificate number SYXK(E)2022‐0061). The rats had unrestricted access to food and water and were housed in specific pathogen‐free environments at 25±3 °C and 50–60% humidity, with a 12‐h light‐dark cycle. The rats were acclimated for one week before the establishment of burn models.

### Burn Injury Model

A third‐degree burn covering 30% of TBSA was induced in the burn group by anesthetizing and shaving the rats, then immersing their backs in water at 96 °C for 20 s. The sham group underwent similar procedures using water at 36 °C. Post‐burn, the rats received warmed saline (5 mL per 100 g body weight) and were housed individually. On day four post‐burn, samples of the TA, EDL, GAS, SO muscles, liver, spleen, colon, blood, and feces were collected and stored at −80 °C until analysis.

### Antibiotic Pseudaseptic Burn Model

Prior to establishing the burn model, the rats were given a cocktail of antibiotics (ampicillin, metronidazole, neomycin, and vancomycin) in their drinking water for one week. Fecal samples were collected for 16S rRNA analysis to confirm antibiotic efficacy. Subsequently, the rats were assigned to either the antibiotic‐treated burn (abx‐burn) or antibiotic‐treated sham (abx‐sham) groups. Samples were collected on day four post‐burn.

### Fecal Microbiota Transplantation Burn Model

Fecal samples were collected from both burn and sham rats during days 1–7, resuspended in PBS, and filtered. Recipient rats underwent a one‐week antibiotic pretreatment before being assigned to either the fecal microbiota transplantation burn (FMT‐burn) or fecal microbiota transplantation sham (FMT‐sham) groups. Each group received fecal suspensions via gastric gavage (10 µL per gram of body weight) and were topically applied to the lower abdomen and back every other day for 30 days. After treatment, fecal samples were analyzed for 16S rRNA, along with tissue samples.

### Palmatine Treatment

Rats were administered palmatine (100 mg kg^−1^) or PBS (10 mL kg^−1^) via gastric gavage daily for 7 days, as previously reported.^[^ ^41^ ^]^ Colonic samples were collected to assess the suppression of IDO‐1 expression. Once confirmed, the rats were assigned to either the palmatine burn (Pal‐burn) or PBS burn (PBS‐burn) groups for third‐degree burns and respective sham models. On day four post‐burn, samples of the TA, EDL, GAS, SO muscles, liver, spleen, colon, blood, and feces were collected.

### Burned Patients and Volunteers

Data collection was approved by the Institutional Ethics Committee of Tongren Hospital, Wuhan University (approval number: KY2023‐014). Between January and December 2023, 20 burned patients were enrolled based on the following criteria: 1) burns covering ≥20% TBSA, classified as “major”;^[^ ^42^ ^]^ 2) age between 20 and 55 years; 3) no history of metabolic diseases (hypertension, diabetes, heart disease, and obesity); 4) no pre‐existing liver or kidney dysfunction; 5) no antibiotic use in the month prior to hospitalization; and 6) signed informed consent. Exclusion criteria included excessive burn areas requiring more than three debridement and skin grafting procedures within a month or severe burns resulting in death.

For the control group, healthy volunteers were selected based on the following criteria: 1) age between 20 and 55 years; 2) body mass index (BMI) between 18.5 and 24 kg m^−^^2^; 3) good health with no liver or kidney diseases, metabolic disorders, or other underlying conditions; 4) no antibiotic use in the past month; and 5) normal blood test results for liver and kidney function. Exclusion criteria included individuals who were overweight or underweight and those unable to cooperate.

Serum was collected from both burned patients and healthy volunteers. Demographic data included age, sex, height, burn area for burned patients, and weight at hospitalization. Some patients remained hospitalized for months or up to a year after discharge due to their conditions. To evaluate skeletal muscle mass, stored computed tomography (CT) images were analyzed, the skeletal muscle area at the third lumbar vertebra (L3) was assessed, which included the psoas muscle, quadratus lumborum, transversus abdominis, external and internal oblique muscles, rectus abdominis, and erector spinae. The L3 skeletal muscle index (L3‐SMI) was calculated by adjusting the area for height.^[^ ^43^ ^]^ Image analysis was performed using SliceOmatic software (version 4.2, TomoVision, Montreal, Canada).

### Special Diets for Rats

Rats were fed a diet supplemented with L‐Tryptophan (Envigo, TD.170745) in addition to a normal diet (Envigo, TD.07788).^[^ ^44^ ^]^ After 7 days, the rats were randomly divided into two groups for a 30% TBSA third‐degree burn model (Trp‐burn and Ctrl‐burn) and a sham model (Trp‐sham and Ctrl‐sham). On the fourth day post‐burn, samples of tibialis anterior (TA), extensor digitorum longus (EDL), gastrocnemius (GAS), soleus (SO) muscle, liver, spleen, colon, and blood were collected.

### L6‐Myoblast Cell Culture

L6 myoblasts (Procell Biotechnology, CL‐0136) were cultured in high‐glucose DMEM (Servicebio, G4511) supplemented with 10% fetal bovine serum (FBS) (Servicebio, G8001) and antibiotics (100 units mL^−1^ penicillin and 100 µg mL^−1^ streptomycin). When cell density reached ≈60%, the medium was replaced with differentiation medium containing 2% horse serum (VivaCell Biosciences, C2510). After 7 days, differentiation into myotubes was confirmed by mRNA expression of MyoD and MyoG.

After differentiation, L‐Kynurenine (Macklin, 2922‐83‐0) was added at final concentrations of 50, 100, and 150 µmol for 24 h. Prior to addition, Kyn was dissolved in DMSO and incubated at 37 °C for three days, as previously reported.^[^ ^45^ ^]^

### Gene Silencing–siRNA Transfection

L6 myotubes were transfected with siRNAs using Lipo8000TM (Beyotime, C0533) according to the manufacturer's protocol. After differentiation, siRNAs were incubated with Lipo8000TM in Opti‐MEM (31985, Thermo Fisher Scientific) for 15 min to form complexes, which were then added to the cells in differentiation medium. The siRNAs used are listed in Table S2 (Supporting Information). After a 6‐h incubation, the cells were treated with 100 µmol Kyn for 24 h.

### Co‐IP Assay

Proteins were harvested after treatment and divided into two portions for Co‐IP and Input assays. Approximately 100 µL of protein lysates were incubated with 20 µL of Protein A/G Agarose (Millipore, IP05) and the appropriate antibodies (HSP90 (Proteintech, 13171‐1‐AP), Cul4B (Proteintech, 12916‐1‐AP), AHR (Proteintech, 28727‐1‐AP)) at 4 °C overnight with gentle shaking. The samples were then centrifuged at 6000 rpm for 3 min and washed five times with 1 mL of pre‐cooled PBS. About 10 µL of 5×SDS was added to each tube, and the samples were heated to 95 °C for 10 min. Finally, the samples were loaded onto a 10% SDS‐PAGE gel for Western blot analysis.

### 16S rRNA Gene Microbiota Profiling

Fecal samples were collected and analyzed for microbial profiling. Bacterial genomic DNA was extracted using the Quant‐iT PicoGreen dsDNA Assay Kit. PCR amplification targeted the V3‐V4 region using barcoded primers. Illumina TruSeq sequencing was performed at Bioyi Biotechnology Co. (Wuhan, China), and 16S rRNA data analysis was conducted using QIIME2.

### LC‐MS for Fecal Metabolomics

Fecal samples were prepared and analyzed using LC‐MS by Bioyi Biotechnology Co. Approximately 100 mg of fecal matter was ground with liquid nitrogen and transferred to an EP tube. Then, 500 µL of 80% methanol‐water was added. The mixture was vortexed, placed in an ice bath for 5 min, and centrifuged at 15 000 rpm for 20 min at 4 °C. The supernatant was diluted with mass spectrometry‐grade water to achieve a methanol concentration of 53%. After a second centrifugation, the supernatant was analyzed using LC‐MS.

### HPLC‐MS/MS for Muscle and Serum Samples

Muscle or serum samples were thawed on ice. For muscle samples, 50 mg was accurately weighed, and for serum samples, 50 µL was measured into a centrifuge tube and homogenized. Next, 500 µL of methanol (containing 20 µL of internal standard solution at 250 ng mL^−1^) was added. The mixture was vortexed for 3 min and then stored at −20 °C for 30 min. After centrifugation at 12 000 rpm for 10 min at 4 °C, 150 µL of the supernatant was transferred into a sample vial for HPLC‐MS/MS analysis.

### H&E Staining

H&E staining was performed on tibialis anterior (TA) muscle to examine histological changes. Representative images were captured, and the cross‐sectional area (CSA) of individual myofibers was measured using Image‐Pro Plus 6.0 software.

### IF Staining

Colon tissues were fixed in 4% paraformaldehyde, embedded in paraffin, sectioned, and stained for IDO‐1 (Proteintech, 13268‐1‐AP). Images were captured to quantify the number of goblet cells per crypt.

### RT‐qPCR Analysis

Total RNA from L6 myotubes or rat tissues was extracted using TRIzol reagent. RNA integrity was assessed by gel electrophoresis. Total RNA was reverse transcribed into cDNA using the SweScript All‐in‐One SuperMix. RT‐qPCR was conducted using Universal Blue SYBR Green qPCR Master Mix, normalizing results to GAPDH with the 2^−ΔΔCt^ method. All primers were sourced from Servicebio Biotechnology Co., Ltd., and the corresponding qRT‐PCR primer sequences are detailed in Table S2 (Supporting Information).

### Western Blotting

L6 myotubes or tissues were lysed and separated using SDS‐PAGE, then transferred to PVDF membranes. The membranes were incubated overnight with primary antibodies: rat IDO‐1 (Proteintech, 13268‐1‐AP), β‐actin (Servicebio, GB12001), MAFbx (Proteintech, 67172‐1‐Ig), MuRF‐1 (Proteintech, 55456‐1‐AP), HSP90 (Proteintech, 13171‐1‐AP), Cul4B (Proteintech, 12916‐1‐AP), AHR (Proteintech, 28727‐1‐AP), CYP1A1 (Proteintech, 13241‐1‐AP), CYP1A2 (Proteintech, 19936‐1‐AP), CYP1B1 (Proteintech, 18505‐1‐AP), AHRR (Servicebio, GB115154), and GAPDH (Proteintech, 10494‐1‐AP). Detection was performed using HRP‐conjugated secondary antibodies. Bands were visualized using ECL reagents, and intensities were quantified with ImageJ.

### ELISA

Serum was collected and analyzed using the Rat IDO‐1 Kit (Bioswamp, RA27662).

### Statistical Analysis

Statistical analyses were conducted using SPSS 22.0 and GraphPad Prism 8.0. Data are presented as means ± SEM. Student's *t*‐test and one‐way ANOVA were employed for comparisons, with *p* < 0.05 considered statistically significant.

## Conflict of Interest

The authors declare no conflict of interest.

## Author Contributions

S.G., Y.L., and Z.Q. contributed equally to this work. S.G., W.X., S.L., and Z.X. designed the research. S.G., Y.L., Z.Q., K.L., J.L., J.P., S.L., and Z.X. performed the experiments. S.G., Y.L., J.L., and J.P., analyzed the data. S.G., Y.L., Z.Q., S.L., and Z.X. wrote the manuscript.

## Supporting information



Supporting Information

## Data Availability

The data that support the findings of this study are available from the corresponding author upon reasonable request.
