# Supplementary material for: Burn‐Induced Gut Microbiota Dysbiosis Aggravates Skeletal Muscle Atrophy by Tryptophan‐Kynurenine Mediated AHR Pathway Activation
Source: Adv Sci (Weinh). 2025 Feb 14;12(14):2409296. doi: 10.1002/advs.202409296 (PMC11984878; doi:10.1002/advs.202409296)
Supplement: Supplementary file 1 — Supporting Information [file ADVS-12-2409296-s001.docx]

**Burn-Induced Gut Microbiota Dysbiosis Aggravates Skeletal Muscle Atrophy by Tryptophan-Kynurenine Mediated AHR Pathway Activation**

Shan Gao^1;2^, Yan Leng^1^, Zhen Qiu^1^, Kai Li^3^, Jun Li^2^, Jian Peng^2^, Weiguo Xie^4^, Shaoqing Lei^1*^, Zhongyuan Xia^1*^

1 Department of Anaesthesiology, Renmin Hospital of Wuhan University, Wuhan, Hubei 430060, China

2 Department of Anaesthesiology, Tongren Hospital of Wuhan University Wuhan, Hubei 430060, China

3 Department of Pain, Tongren Hospital of Wuhan University, Wuhan, Hubei 430060, China

4 Department of Burns, Tongren Hospital of Wuhan University, Wuhan, Hubei 430060, China

*Correspondence to:

Dr. Zhongyuan Xia, MD, Ph. D

Department of Anesthesiology

Renmin Hospital of Wuhan University, Wuhan, Hubei, China

E-mail: [xiazhongyuan2005@aliyun.com](mailto:xiazhongyuan2005@aliyun.com)

Or to

Dr. Shaoqing Lei, MD, Ph. D

Department of Anesthesiology

Renmin Hospital of Wuhan University, Wuhan, Hubei, China

E-mail: [leishaoqing@163.com](mailto:leishaoqing@163.com)

**Figure S1**


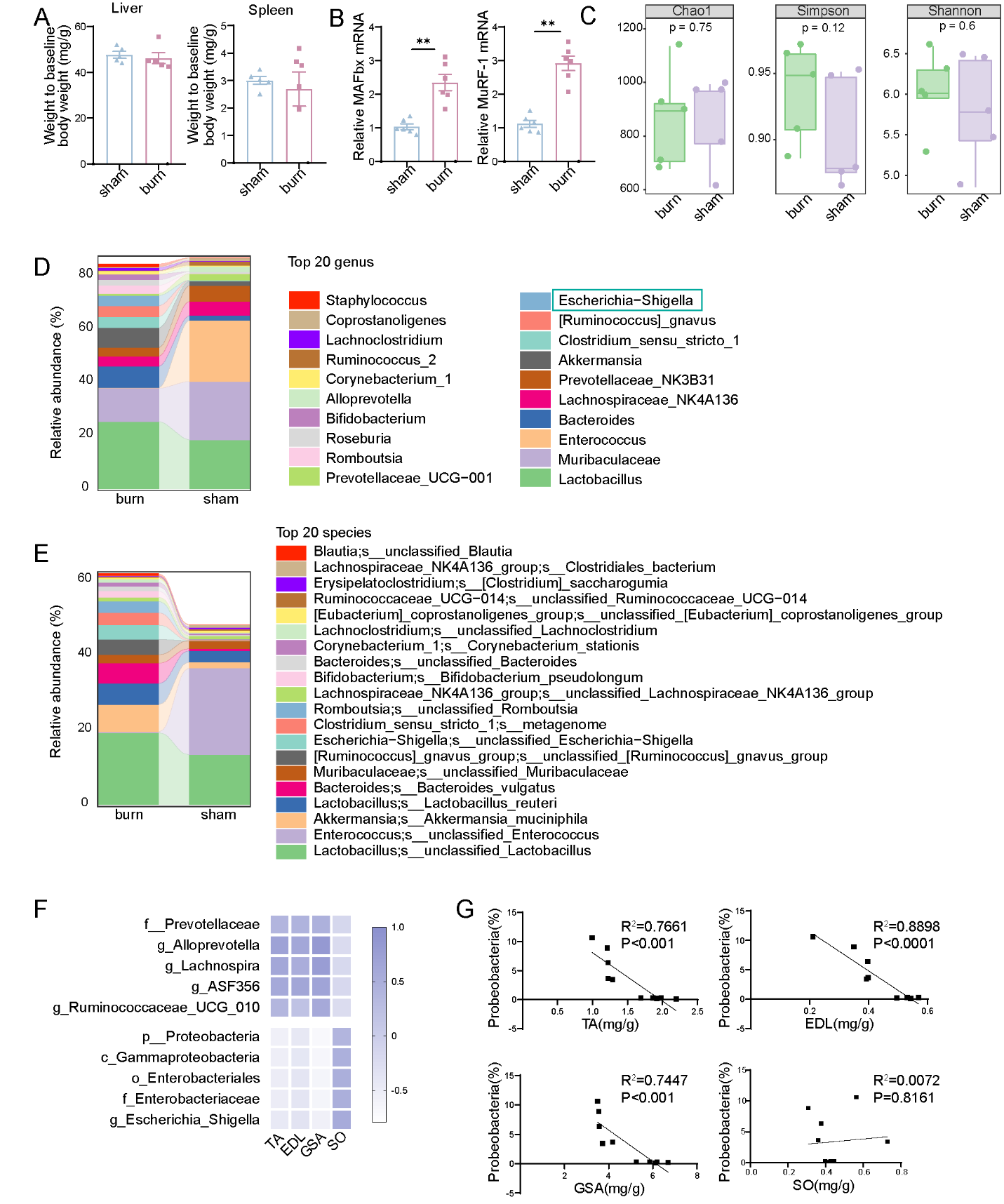


***Supplement Figure S1. Skeletal muscle atrophy is associated with dysbiosis and specific changes in gut microbiota post burn injuries.***

**A,** Relative weight of the liver and the spleen in burn and sham groups.

**B,** Relative mRNA expression of MAFbx and MuRF-1 in the muscle of burn and sham groups.

**C,** α-Diversity analysis based on 16s rDNA profiling of feces from burn and sham groups.

**D,** Relative abundance at genes level in the feces from burn and sham groups.

**E,** Relative abundance at species level in the feces from burn and sham groups.

**F,** Pearson correlation heatmap analysis was performed at the representative LDA >3 microbial genera and skeletal muscle weight (TA/EDL/GAS/ SO), comparing the sham and burn groups.

**G**, Relationships between *Proteobacteria* and skeletal muscle weight (TA/EDL/GAS/ SO) were examined. Pearson correlation coefficient (r) and *P* values are presented.

All the quantitative data (A, B) were analyzed with unpaired two-tailed Student’s *t*-test and are shown as mean ± SEM. ^**^*P* ≤ 0.01.

**Figure S2**


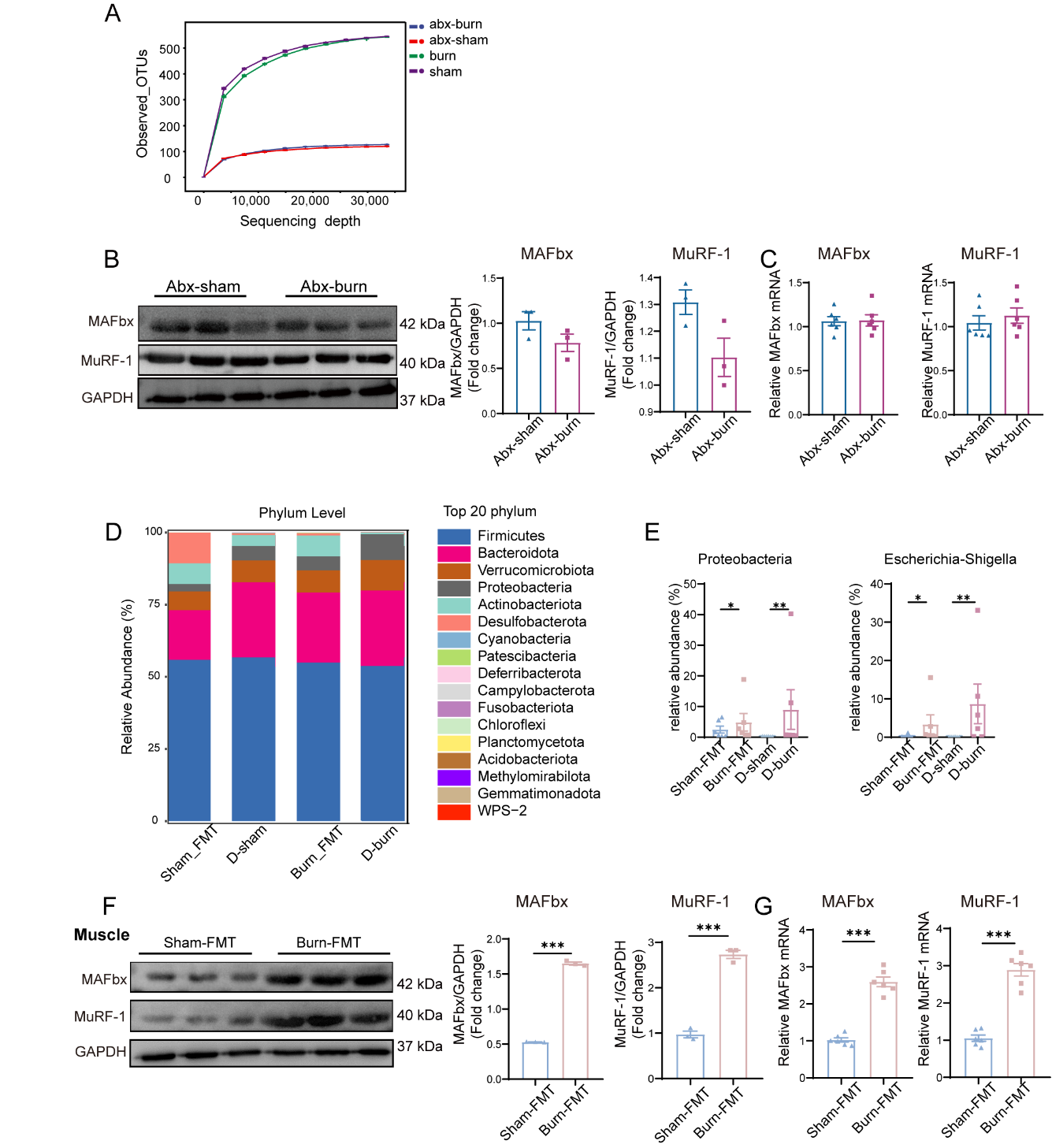


***Supplement Figure S2. Skeletal muscle atrophy is reversed after gut microbiota depletion and transplantation with fecal microbiota of D-burn rats to abx-rats significantly aggravates skeletal muscle atrophy.***

**A,** Observed Operational Taxonomic Unit (OTU) in burn, sham, abx-burn, abx-sham groups.

**B,** Relative MAFbx and MuRF-1 protein expression in muscle from abx-sham and abx-burn groups.

**C,** Relative MAFbx and MuRF-1 mRNA expression in muscle from abx-sham and abx-burn groups.

**D,** Relative abundance at phylum level in the feces from D-burn, D-sham, burn- FMT, and sham-FMT groups.

**E,** Fecal contents of D-burn, D-sham, burn- FMT, and sham-FMT groups were analyzed for *Proteobacteria* and *Escherichia Shigella* colonization by RT-qPCR.

**F,** Relative MAFbx and MuRF-1 protein expression in muscle tissure from burn- FMT, and sham-FMT groups.

**G,** Relative MAFbx and MuRF-1 mRNA expression in muscle tissure from burn- FMT, and sham-FMT groups.

All the quantitative data(B,C,E,F,G) were analyzed with unpaired two-tailed Student’s *t*-test and are shown as mean ± SEM. ^*^*P* ≤ 0.05; ^**^*P* ≤ 0.01; ^***^*P* ≤ 0.001.

**Figure S3**

**
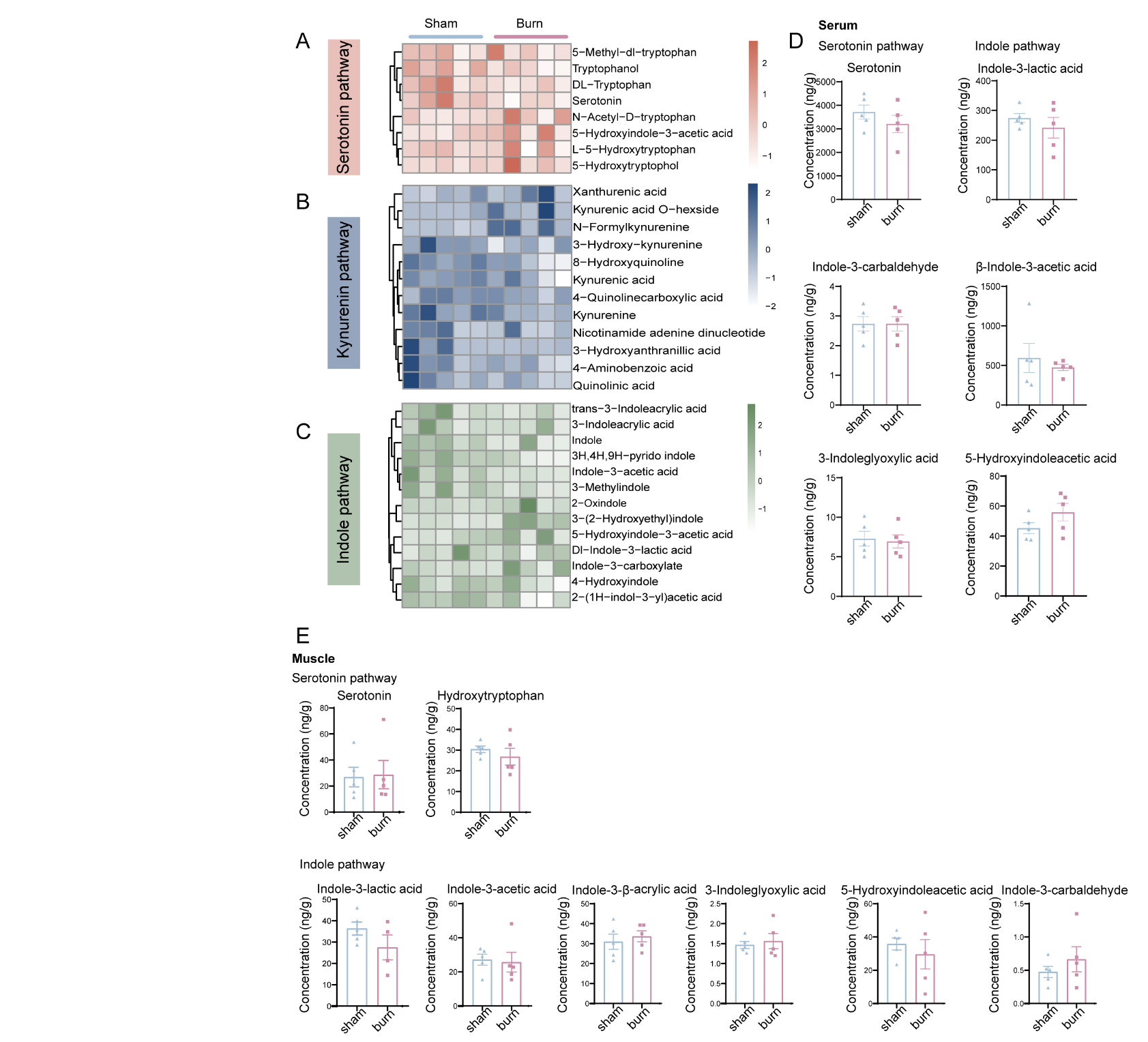
**

***Supplement Figure S3.*** ***Clustered image map of the significantly differential tryptophan-derived metabolic profiles between burn and sham group*.**

**A,** Clustered image map of the serotonin pathway in fecal samples between burn and sham group.

**B,** Clustered image map of the kynurenine pathway in fecal samples between burn and sham group.

**C,** Clustered image map of the aryl hydrocarbon receptor (AHR) ligands indole pathway in fecal samples between burn and sham group.

**D,** Serum tryptophan metabolic profiles in the serotonin pathway and the indole pathway and skeletal muscle between burn and sham group.

**E,** Muscle tryptophan metabolic profiles in the serotonin pathway and the indole pathway and skeletal muscle between burn and sham group.

All the quantitative data (D, E) were analyzed with unpaired two-tailed Student’s *t*-test and are shown as mean ± SEM. ^**^*P* ≤ 0.01.

**Figure S4**

**
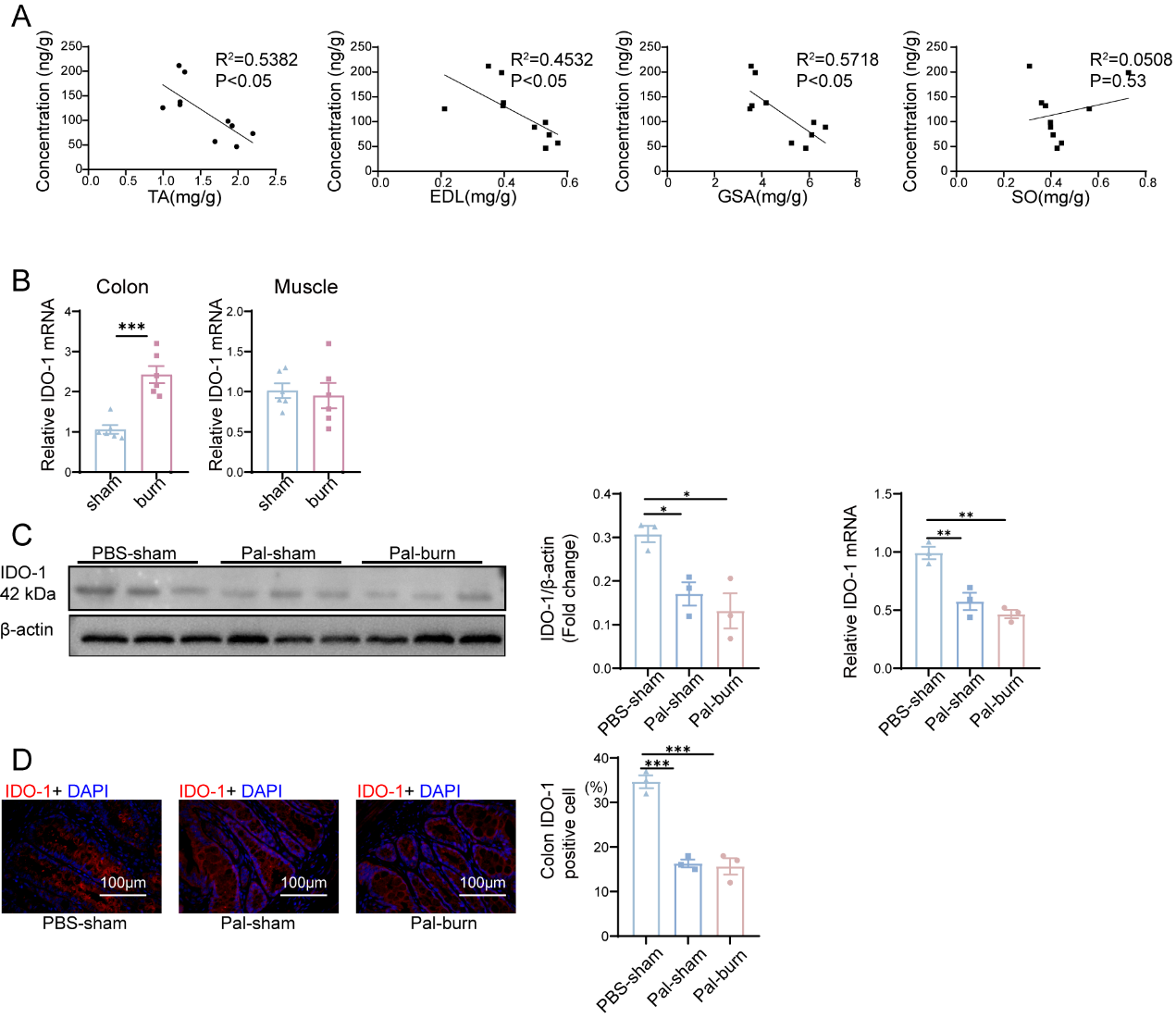
**

***Supplement Figure S4. Colon IDO-1 is effectively inhibited post palmatine treatment.***

**A**, Relationships between muscle Kyn concentration and skeletal muscle weight (TA/EDL/GAS/ SO) were examined. Pearson correlation coefficient (r) and *P* values are presented.

**B**, Relative IDO-1 mRNA expression in the colon and muscle of burn and sham groups.

**C**, Relative IDO-1 protion and mRNA expression in colon from PBS-sham, Pal-sham, and Pal-burn groups.

**D,** IF staining of IDO-1 in colon from PBS-sham, Pal-sham, and Pal-burn groups at 4 days after burn, followed by IDO-1 positive cell analysis. Scale bars, 100 μm.

The quantitative data (B) were analyzed with unpaired two-tailed Student’s *t*-test and are shown as mean ± SEM, data of (C, D) was analyzed with one-way ANOVA. ^*^*P* ≤ 0.05; ^**^*P* ≤ 0.01; ^***^*P* ≤ 0.001.

**Figure S5**

**
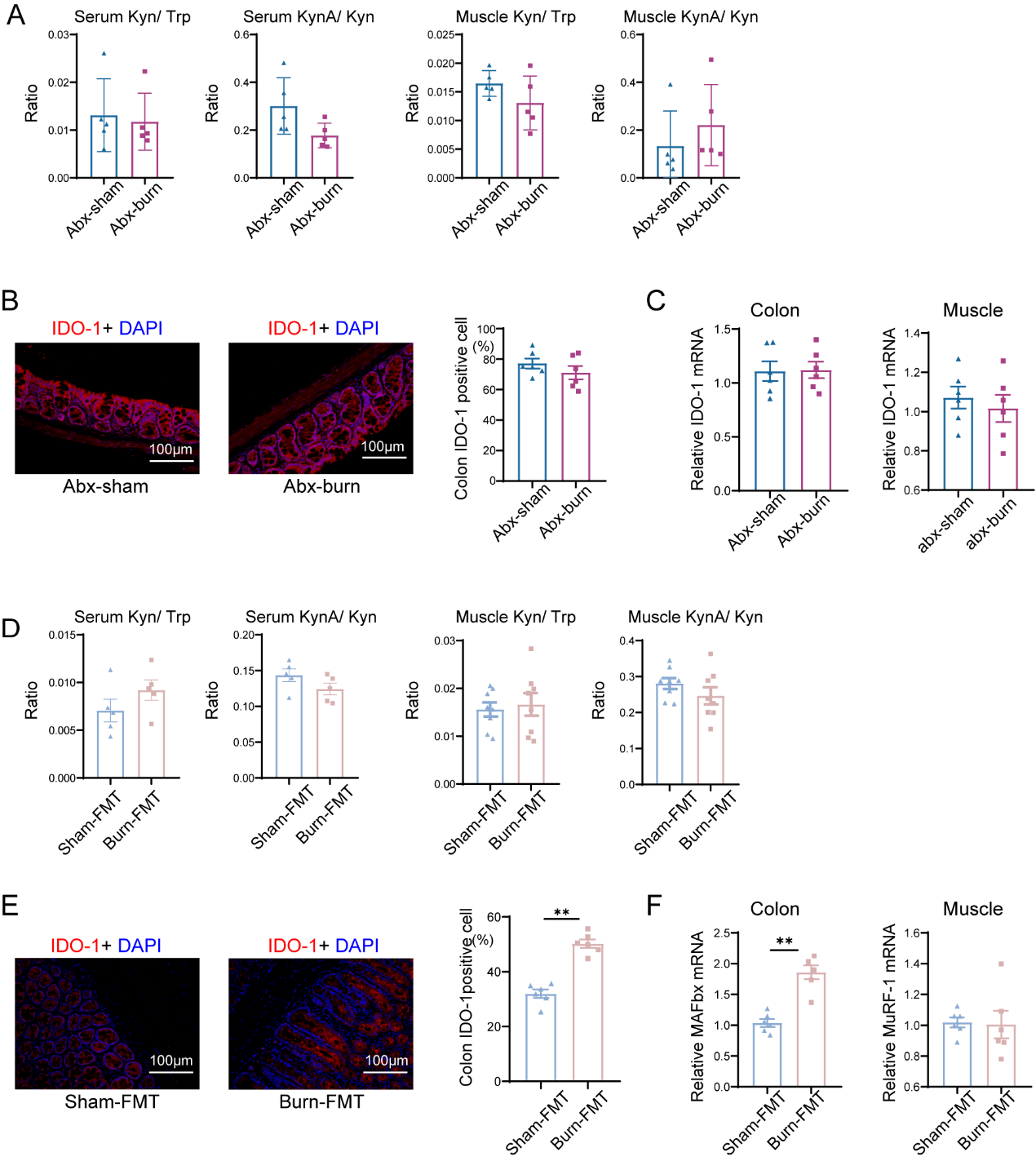
**

***Supplement Figure S5. The expression of colon IDO-1 is closely related to the presence of gut microbiota and affects peripheral Trp-Kyn metabolism.***

**A**, Serum and muscle tissue levels of Kyn/Trp ratio and KynA/Kyn ratio in abx-sham and abx-burn group.

**B**, IF staining of IDO-1 in colon from abx-sham and abx-burn group, followed by IDO-1 positive cell analysis. Scale bars, 100 μm.

**C**, Relative IDO-1 mRNA expression in colon and muscle tissue in abx-sham and abx-burn group.

**D**, Serum and muscle tissue levels of Kyn/Trp ratio and KynA/Kyn ratio in burn- FMT and sham-FMT groups.

**E**, IF staining of IDO-1 in colon from burn- FMT and sham-FMT groups, followed by IDO-1 positive cell analysis. Scale bars, 100 μm.

**F**, Relative IDO-1 mRNA expression in colon and muscle tissue in burn- FMT and sham-FMT groups.

All the quantitative data were analyzed with unpaired two-tailed Student’s *t*-test and are shown as mean ± SEM. ^**^*P* ≤ 0.01.

**Figure S6**


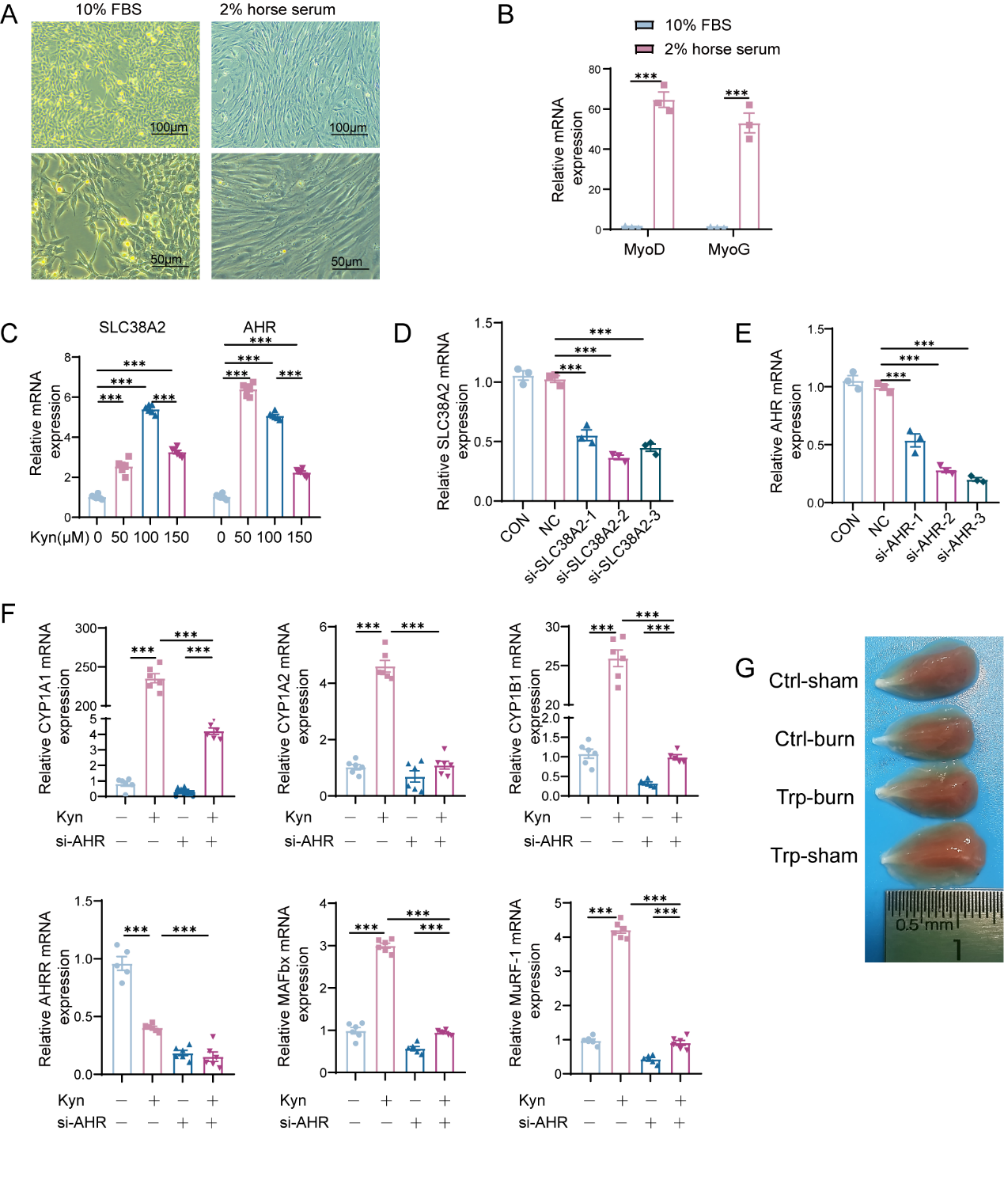


***Supplement Figure S6. Kyn induces proteolysis in L6 myotubes via activation of the AHR pathway, and a Trp-enriched diet ameliorates skeletal muscle atrophy in burned rats.***

**A,** Morphology of L6 cells observed under microscope in the differentiation group (2% horse serum) and the control group (10%FBS). Scale bars, 100 μm and 50 μm.

**B,** Relative MyoD and MyoG mRNA expression in the differentiation group (2% horse serum) and the control group (10%FBS).

**C,** Bar graph showing RT-qPCR quantification of SLC38A2 and AHR gene expression levels in L6 myotubes treated with Kyn (50 μmmol, 100 μmol, 150 μmol) or vehicle for 24h.

**D,** Bar graph showing RT-qPCR quantification of SLC38A2 mRNA expression in L6 myotubes transfected with control (CON), control siRNA (NC-SLC38A2) and three different AHR si SLC38A2s (si-SLC38A2-1, si-SLC38A2-2 and si-SLC38A2-3).

**E,** Bar graph showing RT-qPCR quantification of AHR mRNA expression in L6 myotubes transfected with control (CON), control siRNA (NC- AHR) and three different AHR si AHRs (si-AHR-1, si-AHR-2 and si-AHR-3).

**F,** Bar graph showing RT-qPCR quantification of CYP1A1, CYP1A2, CYP1B1, AHRR, MAFbx, and MuRF-1 gene expression levels in L6 myotubes transfected with AHR siRNAs or vehicle.

**G,** Gross appearance of TAs from the indicated groups.

All the quantitative data were analyzed with one-way ANOVA and are shown as mean ± SEM. ^***^*P* ≤ 0.001.

Table S1. Study population characteristics of patients with burns and health volunteers. Data are expressed as numbers and frequencies for categorical variables and medians and interquartile ranges (25th to 75th percentile).

|  | Patients with burns (n=20) | Volunteers (n=20) | *P* valuea |
| --- | --- | --- | --- |
| Age [years] | 40 (35-47) | 41.5 (39-45) | 0.126 |
| Gender male, n [%) | 13 (65) | 11 (55) | 0.747 |
| Body mass index [kg m^-2^] | 20.3 (16-21) | 22 (18-21) | 0.259 |
| Weight [kg] | 69 (51-72) | 70 (51-76) | 0.862 |
| Height [cm] | 168 (162-170) | 170 (162-175) | 0.581 |
| TBSA [%] | 25 (20-30) | 0 | <0.001 |
| L3-sMI [cm^2^ m^-2^] | 37.39 (33-40)* | 45.54 (43-48)* | <0.001 |

*Record/data were statistics in 8 patients during our study period. Abbreviations: TBSA, total body surface area; L3-sMI, L3 skeletal muscle index.

Table S2. Primers and siRNAs used in the study

| Gene name | Oligonucleotides |
| --- | --- |
| MuRF-1 | F 5’- GTGCCTACTTGCTCCTTGTGC -3’ |
|  | R 5’- GGCGTAGAGGGCGTCAAACT -3’ |
| MAFbx | F 5’- ACATCCCTGAGTGGCATCGC -3’ |
|  | R 5’- CATGTTGATGTTGCCCACCA -3’ |
| SLC38A2 | F 5’-GCAGGCTGCTCTAAAAAGCCAT-3’ |
|  | R 5’-AGGATTCCACTGCCCACAAT-3’ |
| AHR | F 5’-TCAGAGACCGCTAACGGATG-3’ |
|  | R 5’-CTTACTCGGGGTTGACTGGG-3’ |
| CYP1A1 | F 5’-CCTATCCTCCGTTACCTCCCT-3’ |
|  | R 5’-CCGGATGTGGCCCTTCTC-3’ |
| CYP1A2 | F 5’-ATCCCCCACAGCACAACG-3’ |
|  | R 5’-CGATGGCCGTGTTGTCATTG-3’ |
| CYP1B1 | F 5’-TGAGCCAAGAGTGGGTGCTA-3’ |
|  | R 5’-TGGTGGCCATGCTGCG-3’ |
| IDO-1 | F 5’-GACTTCGTGGATCCAGAC-3’ |
|  | R 5’-TCTAAGGAGG AGAGGAAG-3’ |
| AHRR | F 5’-CTTCGCCTCAGCGTGAGTTA-3’ |
|  | R 5’-GCCATTGAGAGACTCCAGCA-3’ |
| si SCL38A2-1 | F 5’-AAAAGUUCUGGUUUUCGGGGU-3’ |
|  | R 5’-CCCGAAAACCAGAACUUUUUA-3’ |
| si SCL38A2-2 | F 5’-AUUCAAGUAAAAAGUUCUGGU-3’ |
|  | R 5’-CAGAACUUUUUACUUGAAUCG-3’ |
| si SCL38A2-3 | F 5’-AGUAGUACCUGGAUGAAAGUC-3’ |
|  | R 5’-CUUUCAUCCAGGUACUACUUC-3’ |
| si SLC38A2-NC | F 5’-AGUCAAGUCGCCGUACUAGUC-3’ |
|  | R 5’-CUUCGUUCGGUAUCGGUAUUC-3’ |
| si AHR-1 | F 5’-ACUCCCUCCACAGUUGGCUUUGUUU-3’ |
|  | R 5’- AAACAAAGCCAACUGUGGAGGGAGU-3’ |
| si AHR-2 | F 5’-GCUGUGAUGCCAAAGGGCAGCUUAU-3’ |
|  | R 5’- AUAAGCUGCCCUUUGGCAUCACAGC-3’ |
| si AHR-3 | F 5’-CCGCAAGCAAUGAAGCUCUGCUGAA-3’ |
|  | R 5’- UUCAGCAGAGCUUCAUUGCUUGCGG-3’ |
| si AHR-NC | F 5’-CCGACGAAGUAUCGACGUCUACGAA-3’ |
|  | R 5’- UUCGUAGACGUCGAUACUUCGUCGG-3’ |
| GAPDH | F 5’- CTGGAGAAACCTGCCAAGTATG -3’ |
|  | R 5’- GGTGGAAGAATGGGAGTTGCT -3’ |
